# Systems Biology Reveals *NR2F6* and *TGFB1* as Key Regulators of Feed Efficiency in Beef Cattle

**DOI:** 10.3389/fgene.2019.00230

**Published:** 2019-03-22

**Authors:** Pâmela A. Alexandre, Marina Naval-Sanchez, Laercio R. Porto-Neto, José Bento S. Ferraz, Antonio Reverter, Heidge Fukumasu

**Affiliations:** ^1^Department of Veterinary Medicine, College of Animal Sciences and Food Engineering, University of São Paulo, Pirassununga, Brazil; ^2^Agriculture and Food, Commonwealth Scientific and Industrial Research Organisation, Brisbane, QLD, Australia

**Keywords:** feed efficiency, residual feed intake, Nellore (Zebu), *Bos indicus*, inflammation, muscle development, motif discovery, regulatory gene network

## Abstract

Systems biology approaches are used as strategy to uncover tissue-specific perturbations and regulatory genes related to complex phenotypes. We applied this approach to study feed efficiency (FE) in beef cattle, an important trait both economically and environmentally. Poly-A selected RNA of five tissues (adrenal gland, hypothalamus, liver, skeletal muscle and pituitary) of eighteen young bulls, selected for high and low FE, were sequenced (Illumina HiSeq 2500, 100 bp, pared-end). From the 17,354 expressed genes considering all tissues, 1,335 were prioritized by five selection categories (differentially expressed, harboring SNPs associated with FE, tissue-specific, secreted in plasma and key regulators) and used for network construction. *NR2F6* and *TGFB1* were identified and validated by motif discovery as key regulators of hepatic inflammatory response and muscle tissue development, respectively, two biological processes demonstrated to be associated with FE. Moreover, we indicated potential biomarkers of FE, which are related to hormonal control of metabolism and sexual maturity. By using robust methodologies and validation strategies, we confirmed the main biological processes related to FE in *Bos indicus* and indicated candidate genes as regulators or biomarkers of superior animals.

## Introduction

Since the domestication of the first species, animal selection aims to meet human needs and their changes over time. The current main selection goals in livestock production are increase of productivity, reduction of the environmental impact and reduction of competition for grains for human nutrition (Hayes et al., 2013). Thus, feed efficiency (FE) has become a relevant trait of study, as animals considered of high feed efficiency are those presenting reduced feed intake and lower production of methane and manure without compromising animal’s weight gain (Gerber et al., 2013). However, the incorporation of FE as selection criteria in animal breeding programs is costly and time consuming. Daily feed intake and weight gain for a large number of animals need to be recorded for at least 70 days to obtain accurate estimates of FE (Archer et al., 1997).

In the past years, several studies have been carried out with the aim to identify molecular markers associated with FE to enable a faster and cost-effective identification of superior animals (Rolf et al., 2011; Oliveira et al., 2014; Santana et al., 2014; Seabury et al., 2017). However, for each population, different biological processes seem to be identified (Rolf et al., 2011; Oliveira et al., 2014; Santana et al., 2014; Seabury et al., 2017). Probably, that is because FE is a multifactorial trait and many different biological mechanisms seems to be involved in its regulation (Herd et al., 2004; Herd and Arthur, 2009). It has been demonstrated that high FE animals present increased mitochondrial function (Connor et al., 2010; Lancaster et al., 2014), less oxygen consumption (Gonano et al., 2014) and delayed puberty (Shaffer et al., 2011; Randel and Welsh, 2013; Fontoura et al., 2016). On the other hand, low FE animals have increased physical activity, ingestion frequency and stress level (Kelly et al., 2010; Cafe et al., 2011; Chen et al., 2014; Francisco et al., 2015), increased leptin and cholesterol levels (Nkrumah et al., 2007; Alexandre et al., 2015; Foote et al., 2016; Mota et al., 2017), higher subcutaneous and visceral fat (Mader et al., 2009; Gomes et al., 2012; Santana et al., 2012), higher energy wastage as heat (Archer et al., 1999; Montanholi et al., 2009, 2010) and more hepatic lesions associated with inflammatory response (Alexandre et al., 2015; Paradis et al., 2015).

In the context of such a complex trait, we perform a multiple-tissue transcriptomic analysis of high (HFE) and low (LFE) feed efficient Nellore cattle across tissues related to endocrine control of hunger/satiety, hydric and energy homeostasis, stress and immune response, physical and sexual activity, as is the case of hypothalamus-pituitary-adrenal axis and organs as liver and skeletal muscle. Using gene co-expression across tissues and conditions, we derived a regulatory network revealing NR2F6 and TGFB1 signaling as key regulators of hepatic inflammatory response and muscle tissue development, respectively. Next, we applied advanced motif discovery methods which (i) validate that co-expressed genes are enriched for NR2F6 and TGFB1 signaling effector molecule SMAD3 binding sites in their 10 kb upstream regions and (ii) predict direct transcription factor (TF) – Target gene (TG) interactions at the sequence level. These binding interactions were experimentally validated with public TF ChIP-seq from ENCODE (Encode Project Consortium, 2012; Sloan et al., 2016). Regulatory activity in the tissues of interest was also confirmed by performing an enrichment analysis on open chromatin tracks and histone chromatin marks across cell types and tissues in the human and cow genome. Moreover, we propose a hormonal control of differences in metabolism and sexual maturity between HFE and LFE animals, indicating potential biomarkers for further validation such as adrenomedullin, FSH, oxytocin, somatostatin and TSH.

## Results

### Multi-Tissue Transcriptomic Data Reveal Differences Between High and Low Feed Efficient Animals

Feed efficiency is a complex trait characterized by multiple distinct biological processes including metabolism, ingestion, digestion, physical activity and thermoregulation (Herd et al., 2004; Herd and Arthur, 2009). To study FE at transcriptional level we performed RNAseq of five tissues (i.e., adrenal gland, hypothalamus, liver, muscle and pituitary) from nine male bovines of high feed efficiency [HFE, characterized by low residual feed intake (RFI) (Koch et al., 1963)] and nine of low FE (LFE, characterized by high RFI). In total, we analyzed 18 samples of liver, hypothalamus and pituitary; 17 of muscle and 15 of adrenal gland, yielding 13 million reads per sample on average (Supplementary Table 1). Gene expression was estimated for 24,616 genes present in the reference genome (UMD 3.1) and after quality control (refer to Section “Materials and Methods”), 17,354 genes were identified as being expressed in at least one of the five tissues analyzed.

Differential expression (DE) analysis between HFE and LFE animals resulted in 471 DE genes across tissues (*P* < 0.001, Supplementary Image 1), namely, 111 in adrenal gland, 125 in hypothalamus, 91 in liver, 104 in muscle and 98 in pituitary (Supplementary Tables 2A–E). Although no significant functional enrichment was found for the 281 genes up-regulated in HFE group, the 248 genes down-regulated presented a significant enrichment of GO terms such as response to hormone (Padj = 5.43 × 10^-6^), regulation of hormone levels (Padj = 3.48 × 10^-6^), cell communication (Padj = 3.18 × 10^-4^), regulation of signaling receptor activity (Padj = 3.20 × 10^-4^), hormone metabolic process (Padj = 5.86 × 10^-4^), response to corticosteroid (Padj = 6.28 × 10^-4^), regulation of secretion (Padj = 7.2 × 10^-4^), response to lipopolysaccharide (Padj = 7.9 × 10^-4^) and regulation of cell proliferation (Padj = 1.86 × 10^-3^). Refer to Supplementary Image 2 to see all enriched terms.

### Overlap Between Gene Selection Criteria Prioritizes Genes Associated With Feed Efficiency

The genetic architecture behind complex traits involves a large variety of genes with coordinated expression patterns, which can be represented by gene regulatory networks as a blueprint to study their relationships and to identify central regulatory genes (Swami, 2009). Therefore, it is important to select relevant genes and gene families according to the phenotype of interest to be used for network analysis. We defined five categories of genes (see Section “Materials and Methods” for further information) for inclusion in co-expression analysis: (1) differentially expressed (DE), (2) genes harboring SNPs previously associated with FE (harboring SNP), (3) tissue specific (TS), (4) genes coding proteins secreted in plasma by any of the five tissues analyzed (secreted) and (5) key regulators.

**FIGURE 1:**
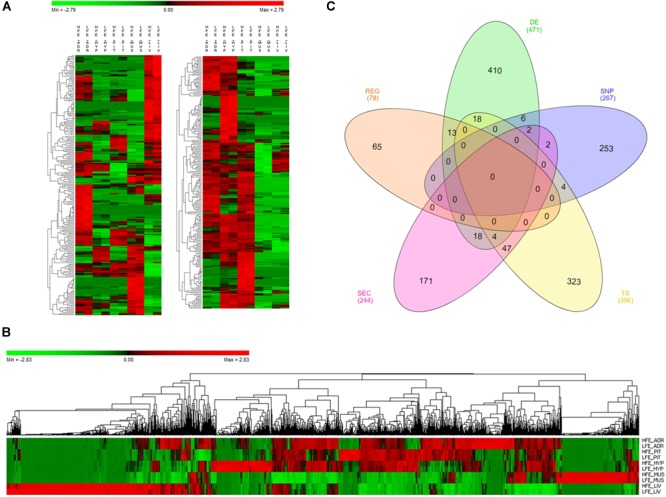
Genes selected for co-expression network construction. **(A)** Heatmap of normalized mean expression (NME) of 471 differentially expressed (DE) genes between high (HFE) and low (LFE) feed efficient animals in adrenal gland (ADR), hypothalamus (HYP), liver (LIV), muscle (MUS) and pituitary (PIT). Genes (rows) and samples (columns) are organized by hierarchical clustering based on Euclidean distances. **(B)** NME heatmap of all 1,335 genes selected for network construction. Genes (columns) and samples (rows) are organized by hierarchical clustering based on Euclidean distances. **(C)** Venn diagram of 1,335 genes selected for network construction. The inclusion criteria for selecting genes were divided into five categories: differentially expressed genes (DE), tissue specific genes (TS), genes harboring SNPs reported in literature as being associated with feed efficiency in beef cattle (SNP), genes encoding proteins secreted by at least one of the tissues in plasma (SEC) and key regulators (REG). Numbers between brackets indicate the total number of genes in each category.

As reported before, we have identified 471 DE genes between HFE and LFE animals (Figure 1A and Supplementary Table 3A). In addition, 267 genes were selected for harboring SNPs previously associated with FE, as not only differences in expression levels can influence the phenotype but also polymorphism in the DNA sequence that can alter the translated protein behavior (Supplementary Table 3B). Moreover, 396 were selected for being tissue specific (refer to Section “Materials and Methods” for definition); 22 in adrenal gland, 32 in hypothalamus, 215 in liver, 118 in muscle and 9 in pituitary (Supplementary Table 3C). A total of 244 genes coding proteins secreted in plasma were selected because of their potential as biomarkers of FE (Supplementary Table 3D). From those, 135 had liver as the tissue of maximum expression and were functionally enriched for GO terms such as complement activation (Padj = 1.82 × 10^-19^), regulation of acute inflammatory response (Padj = 1.89 × 10^-14^), innate immune response (Padj = 9.71 × 10^-12^), negative regulation of endopeptidase activity (Padj = 2.35 × 10^-10^), platelet degranulation (Padj = 1.08 × 10^-10^), regulation of coagulation (Padj = 3.39 × 10^-9^), triglyceride homeostasis (Padj = 1.23 × 10^-6^), cholesterol efflux (Padj = 1.03 × 10^-5^) (Supplementary Image 3). Finally, from 1570 potential regulators in publicly available Animal TFdb, 78 were identified as key regulators of the genes selected by all the other categories, i.e., 78 genes presented a coordinated expression level with many of the genes in the network reflecting a tight control of expression patterns across tissues (Supplementary Table 3E).

Considering all the inclusion criteria, 1,335 genes were selected to be included in co-expression network analysis (Figure 1B and Supplementary Table 4), some of them selected in more than one category (Figure 1C). Regarding DE genes, six of them were also reported before as harboring SNPs associated with the phenotype (*LUZP2, MAOB, SFRS5, SLC24A2, SOCS3* and *WIF1*) (Bolormaa et al., 2011; Saatchi et al., 2014; Ramayo-Caldas et al., 2018) and 13 of them were key regulators (*HOPX, PITX1, CRYM, PLCD1, ND6, CYTB, ND1, MT-ND4L, ND5, ATP8, ND4, ENSBTAG00000046711* and *ENSBTAG00000048135*). Many of the genes that are both DE and regulators are involved in respiratory chain (*ND6, CYTB, ND1, MT-ND4L, ND5, ATP8* and *ND4*) and were all up-regulated in HFE group.

Considering both DE and secreted genes, 18 were identified (*NOV, SPP1, CTGF, OXT, PTX3, VGF, CCL21, COL1A2, PGF, SOD3, SERPINE1, PRL, PON1, SST, JCHAIN, PCOLCE, IGFBP6* and *SCG2*). In addition, four genes were DE, secreted and tissue specific, two from liver (*CXCL3* and *IGFBP1*) and two from pituitary (*NPY* and *CYP17A1*). Genes *RARRES2* and *PENK* (proenkephalin) were DE, secreted and had been previously reported as harboring SNP associated with FE (RARRES2:AnimalQTLdb Release 35 – QTL:20671, rs133399845; PENK: Bolormaa et al. (2011)- rs136198266, rs134428213, rs137492938, rs132881564). Other DE genes worthy to highlight, due to their well-known role in metabolic processes, are *AMH* (anti-mullerian hormone), *TSHB* (thyroid stimulating hormone beta), *FGF21* (Fibroblast growth factor 21) and *FST* (follistatin), up-regulated in HFE group, and *PMCH* (pro-melanin concentrating hormone), *ADM* (adrenomedullin) and *FSHB* (follicle stimulating hormone beta), up-regulated in LFE group.

### Co-expression Network Reveals Regulatory Genes and Biological Processes Related to Feed Efficiency

**FIGURE 2:**
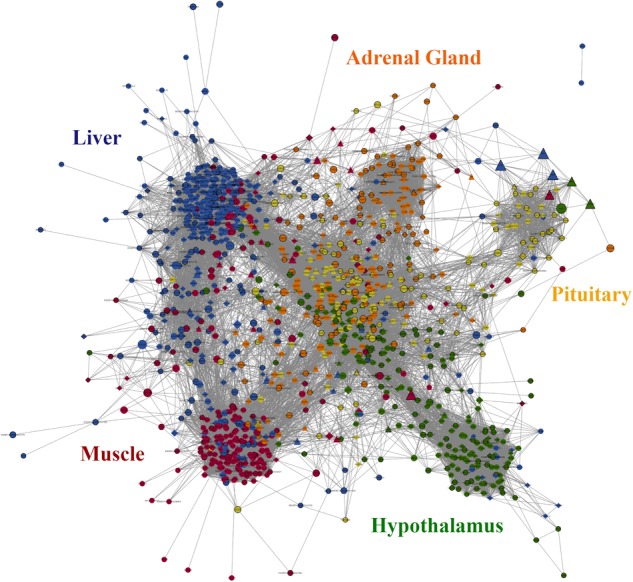
Gene co-expression network constructed using PCIT algorithm on 1,335 selected genes (see Section “Materials and Methods”). Only significant correlations above | 0.9| and their respective genes were considered, totaling to 1,317 genes and 91,932 connections. Nodes with diamond shape correspond to genes coding for proteins secreted in plasma and triangles correspond to key regulators; all the other genes are represented by ellipses. Nodes with black borders are differentially expressed between high and low feed efficiency groups. Colors are relative to the tissue of maximum expression: blue represents liver, red represents muscle, yellow represents pituitary, green represents hypothalamus and orange represents adrenal gland. The size of the nodes is relative to the normalized mean expression values in all samples.

The co-expression network (Figure 2) was composed of 1,317 significant genes and 91,932 connections, with a mean of 70 connections per gene (considering only genes with significant expression correlation ≥|0.90|). Most of the connections (51%) involved a DE gene and 23% of those were between two DE genes. Tissue specific (TS) genes were involved in 49% of the connections with 119 connections per gene on average, which was higher than the overall network mean and reflects the close relationship among genes involved in tissue specific functions. Key regulators were the least represented category in the network (only 78 genes) but accounted for 11% of the connections in the network with the highest value of mean connections per gene, 131 connections, which is in accordance with their regulatory role. Regarding the connections within tissues, when we ranked all the genes in the network by the number of connections and looked at the top 50 genes, 29 were from liver, 15 were from muscle and 3, 2 and 1 were from pituitary, adrenal gland and hypothalamus, respectively. These results indicate very well-coordinated expression patterns in liver and muscle that could be a reflection of the number of TS genes in those tissues and the presence of central regulatory genes coordinating the expression of many other genes.

In the network (Figure 2), genes were grouped together by tissue which was mostly driven by TS genes. As mentioned before, most of the secreted protein-coding genes were located in the liver. Most of the key regulators were located peripherally in relation to the clusters which could be reflecting their regulatory nature independent of tissue specificity. Despite that, some regulators draw attention because of their high number of connections.

The top five most connected regulators were *EPC1, NR2F6, MED21, ENSBTAG00000031687* and *CTBP1*, varying from 317 to 284 connections. They were all first neighbors of each other and were connected mainly to genes with higher expression in liver and essentially enriched for acute inflammatory response (Padj = 4.5 × 10^-13^, Supplementary Image 4). The next most connected regulator is *TGFB1* with 217 connections. It is mainly connected to genes from muscle that are primarily enriched for muscle organ development (Padj = 6.87 × 10^-5^) and striated muscle contraction (Padj = 1.39 × 10^-5^, Supplementary Image 5). Besides indicating main regulator genes, the gene co-expression networks approach can be useful to access the role of specific genes. For instance, gene *FGF21*, a hormone up-regulated in liver of HFE animals, is directly connected to genes enriched for plasma lipoprotein particle remodeling, regulation of lipoprotein oxidation and cholesterol efflux (Padj = 5.64 × 10^-3^, Supplementary Image 6). Indeed, according to the literature, this gene is associated with decrease in body weight, blood triglycerides and LDL-cholesterol (Cheung and Deng, 2014).

### Motif Discovery Confirms NR2F6 as a Key Regulator of Liver Transcriptional Changes Between High and Low Feed Efficiency

By means of the power-law theory, co-expression networks present many nodes with few connections and few central nodes with many connections (de la Fuente, 2010), being the last ones indicated as central regulatory genes responsible for the transcriptional changes between the divergent phenotypes analyzed. In our study, the most connected regulators were indicated, together with their target genes, i.e., their first neighbors in the network. Those genes are a mixture of direct and indirect regulator targets. In order to validate the regulatory role of the most connected regulators in the network and identify their core direct targets, we performed motif discovery in their co-expressed target genes. It is noteworthy that motif discovery should confirm the presence of DNA motifs of a TF in the regulatory regions of co-expressed genes. From the top five most connected regulators from our previous co-expression analysis, only NR2F6 has the ability to bind DNA. In contrast, the other four regulators act mainly as cofactors (corepressor, i.e., CTBP1; coactivator, i.e., MED21; or histones modifier, i.e., EPC1), that is co-binding through protein–protein interactions.

**FIGURE 3:**
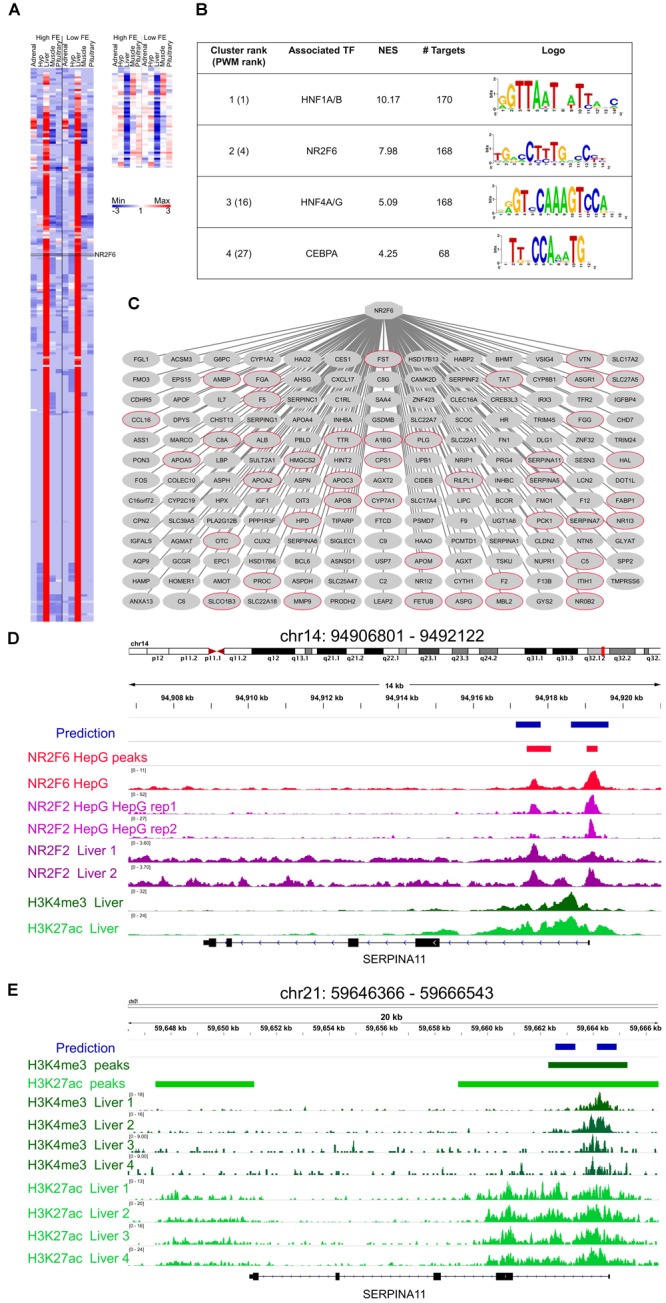
Mapping of NR2F6 direct targets. **(A)** Heatmap of the 313 genes co-expressed with NR2F6 across all samples (derived from the co-expression analysis), **(B)** i-Regulon motif discovery results on the genes shown in panel **(A), (C)** Predicted NR2F6 targetome. A red node indicates genes known to be targeted by NR2F6 in human Hepatocytes. **(D)** Example of predicted NR2F6 target regions for *SERPINA1* gene. The predicted enhancer overlaps the exact position for NR2F6 and NR2F2 binding in HepG sites from the ENCODE dataset as well as histone chromatin marks related to active regulatory regions, namely H3K27ac, and promoters, H3K4me3 in human primary tissue from RoadMap Epigenetics **(E)** The enhancer prediction in cow coordinates (bosTau6) overlaps a region marked with H3K4me3 in cow liver (Villar et al., 2015).

The analysis of 313 co-expressed genes with *NR2F6* (Figure 3A) yield the Nuclear Factor motif HNF4-NR2F2 (transfac_pro-M01031) as the second motif most enriched out of 9732 PWMs (position weight matrices) with a Normalized Enrichment Score (NES) of 7.98 (Figure 3B). In addition, a total of 19 motifs associated with HNF4-NR2F2 were enriched in the dataset, associating HNF4-NR2F2 to 168 direct target genes (Figure 3C). Due to motif redundancy or highly similarity between a plethora of TFs, these motifs can be associated with multiple TFs from HNF4 (direct) to several nuclear factors such as NR2F6 (motif similarity score FDR 1.414 × 10^-5^). However, our co-expression analysis strongly indicates that NR2F6 is the key TF, since it was the TF with the highest number of nodes in the co-expression network (Figure 3C) and neither HNF4 nor NR2F2 were prioritized by any selection category to be included in the network.

Each of the NR2F6 inferred direct target genes contain one or more predicted enhancers, i.e., regions with high-scoring motif binding sites for NR2F6 or TFs with highly similar motifs. To validate the binding of these genomic regions by NR2F6 or TFs with highly similar motifs to NR2F6, we performed a region enrichment analysis of our predicted NR2F6 binding sequences against public TF ChiP-seq bound regions in human cell lines from the ENCODE consortium (1394 TF binding site tracks, Encode Project Consortium, 2012; Sloan et al., 2016). This analysis confirms the experimental binding of TFs with similar binding as NR2F6 in HepG2 cells. In particular, HNF4A on human HepG2 (ENCFF001UGH, GSM803460, NES = 9.57), HNF4G (ENCFF001UGI, GSM803404, NES = 7.83), RXRA (ENCFF001UHJ, GSM803404, NES = 6.85), and NR2F2 (ENCFF001UGV, GSM1010810, NES = 4.45,) as the most enriched tracks (Supplementary Data Sheet 1). Recent NR2F6 ChIP-seq data on HepG2 (ENCODE experiment ENCSR518WPL, GSE96210) also confirms an enrichment for NR2F6, indicating predicted *NR2F6* binding regions are experimentally bound by NR2F6 in hepatocyte cell lines (Figure 3D).

Next, to validate that the NR2F6 binding in those regions is functional in liver we performed an enrichment analysis for open-chromatin (tracks = 655) and histone modifications (tracks = 2450) related to active regulatory elements (Supplementary Table 5). This analysis yielded DNA-seq on human hepatocytes (ENCFF001SOV, GSM816663, NES = 4.10), and H3K29ac and H3K4me3 in adult liver (Roadmap Epigenomics Consortium et al., 2015; GSM621630, GSM537709, respectively) as the most enriched tracks, respectively, strongly indicating that not only predicted target enhancers are bound by NR2F6 in Hepatocyte cell lines, but these regulatory regions are functionally active in hepatocytes and human liver (Figure 3D).

Regarding the cow genome, a recent open-chromatin study (Villar et al., 2015) has mapped active promoters and enhancers by H3K4me3 and H3K27ac ChIP-seq in cow liver resulting in 13,796 promoter and 45,786 enhancers. We performed an enrichment analysis of predicted *NR2F6* enhancers converted to cow coordinates (*n* = 779, Supplementary Table 6, Array Express Accession number E-MTAB-2633) resulting in 446 regions being identified as functional regulatory regions in cow liver. This number is significantly higher compared to the only 43 regions expected to overlap by random (1000 permutation tests) (Figure 3E).

Finally, in addition to *NR2F6* motif, *HNF1A* motif was found as a potential co-regulator in liver, in particular swissregulon-HNF1A.p2 with a NES = 10.17 and in total 20 enriched motifs and 170 direct targets were associated to *HNF1A* (Figure 3B). *HNF1* is a master regulator of liver gene expression (Tronche and Yaniv, 1992), thus making its finding justified.

### Motif Discovery Validates TGFB1 Signaling Through SMAD3/MYOD1 Binding as Drivers of Transcriptional Differences in Muscle of Divergent Feed Efficient Cattle

**FIGURE 4:**
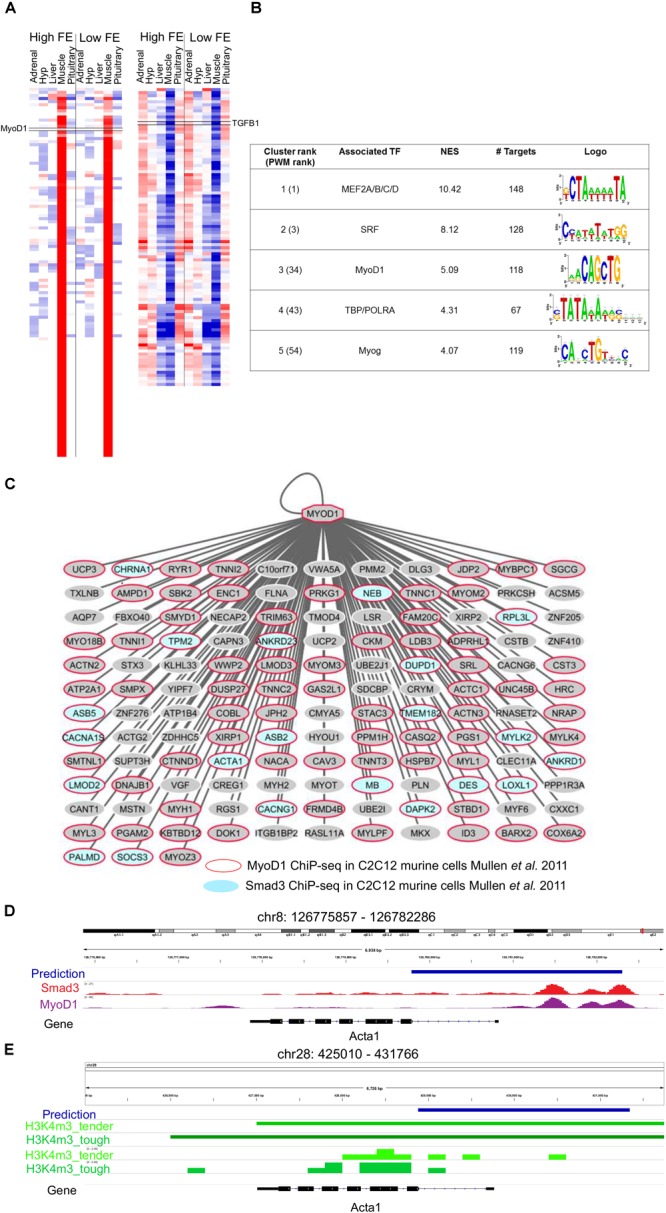
Mapping the downstream network of TGFB1 signaling through SMAD3/MyoD1 DNA binding. **(A)** Heatmap of the 217 genes co-expressed with *TGFB1* (derived from the co-expression analysis). **(B)** i-Regulon motif discovery results on the genes shown in panel **(A), (C)** Predicted MyoD targetome. A red node indicates genes know to be targeted my MyoD1 in murine myotubes (Mullen et al., 2011). Blue nodes indicate genes to be targeted by SMAD3, the effector DNA binding molecular of *TGFB1* signaling, in murine myotubes (Mullen et al., 2011). **(D)** Example of predicted MyoD1 target regions for *ACTA1* gene. The predicted enhancer overlaps the exact position for SMAD3 and MyoD1 ChIP-seq binding in murine myotubes (Mullen et al., 2011). **(E)** The enhancer prediction in cow coordinates (bosTau6) overlaps a promoter region marked with H3K4me3 in muscle tissue in cow (Zhao et al., 2015).

The analysis of the 217 genes co-expressed with TGFB1 (Figure 4A) showed that most target genes motifs were enriched for master regulators of muscle differentiation, namely, MEF2 (NES = 10.42), a MADS box Transcription factor with 148 target genes, and MYOD1 (NES = 5.09), a bHLH transcription factor (CANNTG) with 136 direct target genes (Figure 4B and Supplementary Data Sheet 2). To evaluate the precision of our predicted MYOD1 (bHLH) target genes, we assessed how many of these TF-TG relationships had been previously experimentally reported. Based on MYOD1 ChIP-seq binding in mouse myotubules, 86 genes had already been associated with MYOD1 resulting in a 63% success rate (hypergeometric test 1.72 × 10^-22^). SMAD3, the effector molecule of TGFB1 signaling, is known to recruit MYOD1 to drive transcriptional changes during muscle differentiation (Mullen et al., 2011). Thus, we evaluated whether predicted MYOD1 target genes were enriched for known SMAD3 target genes resulting in 21 out of 135 MYOD1 predicted target genes presenting SMAD3 ChIP-seq binding in myotubes, thus indicating that there is a statistically significant association between MYOD1 target genes and SMAD3 target genes in myotubes (hypergeometric test 1.98 × 10^-6^) (Figure 4C) (Mullen et al., 2011). By contrast, no significant association was found between predicted MYOD1 target genes in this study and SMAD3 target genes in other cell lines, such as pro-B and ES cell (hypergeometric test 0.056 and 0.076, respectively) (Mullen et al., 2011). That is in agreement with the fact that the effect of TGFB1 signaling driven by SMAD3 DNA binding is tissue-specific (Liu et al., 2001). Our analysis predicted 621 potential MYOD1 binding sites, of which 114 (18%, Supplementary Tables 7, 8) and 152 (24.5%, Supplementary Table 9) present a MYOD1 ChIP-seq signal in mouse C2C12 myotubes cells (Mullen et al., 2011) and in primary myotubes (Cao et al., 2009), respectively.

Finally, we evaluate whether predicted MYOD1 binding regions were regulatory regions active in muscle cells across different species, namely human, mouse and cow. To tackle this issue we performed an enrichment analysis across 2113 open-chromatin ENCODE tracks (Encode Project Consortium, 2012; Sloan et al., 2016). This analysis resulted in a clear enrichment of our predicted MYOD1 binding regions with H3K27ac (NES = 15.98) and H3K9ac (NES = 8.78) regions in the skeletal muscle (Figure 4D). Both chromatin marks are associated with active transcription, H3K27ac related to active enhancers and H3K9ac related to active gene transcription (Shin et al., 2012), thus validating most of our enhancer predictions that MYOD1 in human is active in the skeletal muscles. In cow, we assessed the overlap of predicted MYOD1 enhancers and promoter regions in cow muscle experimentally detected with H3K4me3 (Zhao et al., 2015). This resulted in 275 regions out of 653 (42%) overlap when only 11 regions are expected to overlap by random 1000 permutation test) (Figure 4E, Supplementary Table 10).

### Differential Co-expression

Although the general co-expression network provides important insights about regulatory genes and their behavior, by creating specific networks for HFE and LFE and comparing the connectivity of the genes in each one, we can identify genes that change their behavior depending on the situation, moving from highly connected to lowly connected and vice-versa. We were able to identify 87 differentially connected genes between HFE and LFE (*P* < 0.05); 63 mainly expressed in liver, 19 in muscle and 3, 1 and 1 in hypothalamus, adrenal gland and pituitary, respectively (Supplementary Table 11). Those genes were enriched for terms such as regulation of blood coagulation (Padj = 3.14 × 10^-10^), fibrinolysis (Padj = 7.71 × 10^-7^), platelet degranulation (Padj = 7.49 × 10^-6^), regulation of peptidase activity (Padj = 6.16 × 10^-4^), antimicrobial humoral response (Padj = 2.49 × 10^-3^), acute inflammatory response (Padj = 2.18 × 10^-4^) and induction of bacterial agglutination (Padj = 3.58 × 10^-2^) (Supplementary Image 7). It is important to highlight that 20 of the differentially connected genes were also differentially expressed (Table 1) and three of them, i.e., *SST, JCHAIN* and *IGFBP1*, were secreted in plasma as well, which make them very promising potential biomarkers.

**Table 1:** Differentially connected and differentially expressed genes between high and low feed efficiency.

Gene name	Number of connections		Category^∗^	Tissue of maximum expression	Tissue of differential expression
	Low feed efficiency	High feed efficiency			
*SST*	0	45	DE, SEC	Hypothalamus	Hypothalamus
*SNORA73*	41	108	DE	Liver	Liver
*ENSBTAG00000047700*	56	111	DE	Liver	Liver
*ENSBTAG00000047121*	62	111	DE	Liver	Liver
*ENSBTAG00000047816*	53	96	DE	Liver	Liver
*ENSBTAG00000039928*	50	89	DE	Liver	Liver
*ANXA13*	115	63	DE	Liver	Liver
*FST*	113	56	DE	Liver	Liver
*PBLD*	115	55	DE	Liver	Liver
*ENSBTAG00000021368*	95	0	DE	Liver	Liver
*JCHAIN*	52	113	DE, SEC	Liver	Liver
*IGFBP1*	55	0	DE, TS, SEC	Liver	Liver
*SBK2*	0	70	DE	Muscle	Muscle
*ACTC1*	54	0	DE	Muscle	Muscle
*MYH1*	0	47	DE, TS	Muscle	Muscle
*HR*	119	50	DE	Pituitary	Muscle
*TAGLN*	83	31	DE	Adrenal	Muscle, Pituitary
*SFRP2*	41	91	DE	Hypothalamus	Pituitary
*FN1*	119	69	DE	Liver	Pituitary
*CAV1*	98	50	DE	Muscle	Pituitary

*^∗^Differentially expressed genes between high and low feed efficiency (DE), tissue specific genes (TS) and genes encoding proteins secreted in plasma (SEC).*

## Discussion

Feed efficiency is a complex trait, regulated by several biological processes. Thus, the identification of genomic regions associated with this phenotype, as well as regulators genes and biomarkers to select superior animals and to direct management decisions, is still a great challenge. In this work, multi-tissue transcriptomic data of high and low feed efficient Nellore bulls were analyzed through robust co-expression network methodologies in order to uncover some of the biology that governs these traits and put forward candidate genes to be the focus of further research. In this sense, the validation of target genes of main transcription factors (key regulators) in the network by motif search proves the efficacy of the methodology for network construction and prioritizes some transcription factors as central regulators (Aerts et al., 2010; Naval-Sańchez et al., 2013; Potier et al., 2014). Moreover, the addition of a category of genes coding proteins secreted in plasma in the co-expression analysis highlights the genes with potential to be explored as biomarkers of feed efficiency. We were able to identify genes related to main biological processes associated with feed efficiency and indicate key regulators.

Firstly, it is important to state that the 98 animals used to select the HFE and LFE groups in this study have been previously analyzed with regard to several phenotypic and molecular measures (Alexandre et al., 2015; Mota et al., 2017; Novais et al., 2019). It was observed that HFE and LFE groups had similar body weight gain, carcass yield and loin eye area but LFE animals had higher feed intake, greater fat deposition, higher serum cholesterol levels, as well as hepatic inflammatory response, indicated by transcriptome analysis of liver biopsy and proved by the higher number of periportal mononuclear infiltrate (histopathology) and increased serum gamma-glutamyl-transferase (GGT, a biomarker of liver injury) in this group (Alexandre et al., 2015). In the present study, the simultaneous analysis of five distinct tissues revealed the importance of hepatic tissue. Liver presented the most connected genes in the network, the largest number of differentially connected genes and the largest number of secreted genes, which, although can be explained by its biological function, are enriched mostly for terms related to lipid homeostasis and inflammatory response. Moreover, the top five most connected regulators in the network are co-expressed mainly with genes highly expressed in liver and also enriched for inflammatory response.

The relationship between FE and genes or pathways related to immune response and lipid metabolism is becoming more evident, as recent studies also reported in beef cattle (Karisa et al., 2014; Paradis et al., 2015; Weber et al., 2016; Zarek et al., 2017; Mukiibi et al., 2018) and pigs (Gondret et al., 2017; Ramayo-Caldas et al., 2018). In our previous work (Alexandre et al., 2015), we proposed that increased liver lesions associated with higher inflammatory response in the liver of LFE animals could be due to increased lipogenesis and/or higher bacterial infection in the liver. While further evidence is needed to test these hypotheses, the enrichment of terms such as induction of bacterial agglutination and response to lipopolysaccharide makes bacterial infection a strong possibility. Indeed, pigs with low FE were reported to have a increased risk of intestinal inflammation, higher neutrophil infiltration biomarkers and increased serum endotoxin (lipopolysaccharide and other bacterial products) which could be related to increased bacterial infection or to decreased capacity to neutralize endotoxins (Mani et al., 2013). The authors hypothesized that differences in bacterial population could partially explain the increase in circulating endotoxins, which could also be true for cattle given that differences in intestinal and ruminal bacterial population between high and low FE animals have already been reported (Myer et al., 2015, 2016). Furthermore, the literature reports lipopolysaccharides (LPS) may cause up-regulation of adrenomedullin (*ADM*) hormone (Shindo et al., 1998), an up-regulated gene in LFE individuals as showed here. It was also demonstrated in rats that intravenous infusion of LPS caused up-regulation of *ADM* in ileum, liver, lung, aorta, skeletal muscle and blood vessels (Shoji et al., 1995) whereas in our study, *ADM* presented differential expression in muscle, but not in liver.

Against pathogen invasion, a tightly regulated adaptive immune response must be triggered in order to allow T lymphocytes to produce cytokines or chemokines and B cells to differentiate and produce antibodies (Hermann-Kleiter and Baier, 2014). This regulation is known to be strongly influenced by the expression level and transcriptional activity of several nuclear receptors, including the NR2F-family, which consists of three orphan receptors: NR2F1, NR2F2 and NR2F6 (Hermann-Kleiter and Baier, 2014). Those receptors present highly conserved DNA and ligand binding domains among each other and across species (Pereira et al., 2000), and all three are expressed in adaptive and immune cells (Hermann-Kleiter and Baier, 2014). In our study, NR2F6 appeared as the second most connected regulator gene in the network while the other family members, although present in our expression data, were not selected by any of our inclusion criteria, thus indicating they might not be so relevant in our conditions. Indeed, NR2F6 appears to be a critical regulatory factor in the adaptive immune system by directly repressing the transcription of key cytokine genes in T effector cells (Hermann-Kleiter et al., 2008; Klepsch et al., 2016). The role of NR2F6 as a key regulator of inflammatory response in our network was validated at gene level by the identification of the binding motif HNF4-NR2F2 (transfac_pro-M01031) as one of the most enriched in NR2F6 target genes, due to the high similarity between NR2F2 and NR2F6 binding sites. Furthermore, using open chromatin data publicly available, we provided experimental evidence of the binding of TFs with highly similar binding motifs as NR2F6 in hepatocyte cells in humans and in cattle, thus, indicating that predicted target enhancers are functional in this tissue.

Another regulator prioritized in our analysis is *TGFB1*, the sixth most connected gene in the co-expression network, and a potential driver of transcriptional changes between high and low FE cattle in muscle. This gene has been previously described as a master regulator of FE in beef cattle, using genomics and metabolomics data (Widmann et al., 2015). Moreover, our motif discovery analysis showed that *TGFB1* co-expressed genes are mostly enriched for binding sites of master regulators of muscle differentiation such as MEF2 and MYOD. Indeed, public available data show many of TGFB1 target genes were associated with MYOD (Mullen et al., 2011). As it is known, signaling pathways are an effective mechanism for cells to respond to environmental cues by regulating gene expression. TGFB1 signaling triggers the phosphorylation of SMAD2/3 transcription factors, which co-bind with cell-type master regulators at the nuclear level allowing/triggering/leading to cell-type specific transcriptional changes (Schmierer and Hill, 2007; Mullen et al., 2011). In skeletal muscle cells, myoblasts and myotubes, SMAD3 co-binds with MYOD1 (Mullen et al., 2011). The overlap between MYOD1 and SMAD3 target genes demonstrate the significant association between both genes in skeletal muscle, in agreement with the tissue-specific TGFB1 signaling response (Mullen et al., 2011). The overlap percentage between our predicted binding sites and MYOD1 Chip-seq data (18 and 24.5%) confirms previous analyses in mice where they reported only 20% of experimental validated distal enhancers in mouse myotubes with a bHLH (MyoD1) binding were actually bound by MYOD1 ChIP-seq data (Blum et al., 2012). Thus, suggesting that additional transcription-factors and/or histone modifications have a key role in MYOD1 binding. The SMAD3/MYOD1 co-bound regions for known target genes are also captured, such as the promoter regions of *ACTA1* and *ANKRD1*, both genes involved in skeletal muscle differentiation. We also demonstrated predicted MYOD1 binding regions are enriched for muscle regulatory regions across species (human, mouse and cow).

Altogether, we showed that co-expressed genes with *TGFB1* are enriched for SMAD3/MYOD1 binding sites, which we validated at the gene and enhancer level by proving not only MYOD1 and SMAD3 binding, but also their accessibility, in human, mouse and cow. In pigs, increased feed efficiency is associated with stimulation of muscle growth by TGFB1 signaling pathway (Jing et al., 2015). Finally, although not directly co-expressed with *TGFB1*, oxytocin (*OXT*) was DE in muscle and despite the lack of knowledge about its role in this tissue, previous work in cattle showed a massive increase of *OXT* expression in the muscle of bovines chronically exposed to anabolic steroids (De Jager et al., 2011). It is not known yet if oxytocin alone has an anabolic activity, but in a context where muscle growth seems to be associated with high FE animals, this hormone should be the focus of further investigation.

From the 13 regulator genes that are DE between groups, six are involved in respiratory chain and are up-regulated in HFE group. Genes *ND1, ND4, ND4L, ND5, ND6* and also *ND2* (which is DE but not identified as a regulator) are core subunits of the mitochondrial membrane respiratory chain Complex I (CI) which functions in the transfer of electrons from NADH to the respiratory chain, while ATP8 is part of Complex V and produces ATP from ADP in the presence of the proton gradient across the membrane. Interestingly, greater quantities of mitochondrial CI protein were associated with high FE cattle by Ramos and Kerley (2013) whereas Davis et al. (2016) found higher CI-CII and CI-CIII concentration ratios for the same group. Other studies demonstrated that HFE animals consume less oxygen (Chaves et al., 2015) and present lower plasma CO_2_ concentrations, which suggests a decreased oxidation process (Gonano et al., 2014). In general, the literature suggests mitochondrial ADP has greater control of oxidative phosphorylation in high FE individuals (Lancaster et al., 2014) and their increased mitochondrial function may contribute to feed efficiency (Connor et al., 2010). In pigs, differences in mitochondrial function were reported when analyzing muscle (Vincent et al., 2015), blood (Liu et al., 2016) and adipose tissue transcriptomes (Louveau et al., 2016). Differences in metabolic rate associated with FE have long been discussed (Herd and Arthur, 2009) and here the hypothesis is corroborated by the up-regulation of *TSHB* in HFE animals, which stimulates production of T3 and T4 in thyroid, thus increasing metabolism. Metabolism is inhibited by *SST*, a down-regulated hormone in this group which was also found to be differentially connected between HFE and LFE.

Examining the DE genes, many hormones can be identified. Hormones are signaling proteins that are transported by the circulatory system to target distant organs in order to regulate physiology. Regarding the relationship between FE and other production traits of economic importance, FSHB, responsible for spermatozoa production by activating Sertoli cells in the testicles (Walker and Cheng, 2005), is up-regulated in LFE group and is inhibited by follistatin (FST), a gene found to be down-regulated in the same group. Moreover, in rats, it has been demonstrated that FSH secretion is stimulated by somatostatin expression, which is up-regulated in LFE animals (Kitaoka et al., 1989). In this scenario, one could argue that selection for high FE delay reproduction traits, something that could be related to the lower fat deposition in this group, as previously observed (Gomes et al., 2012; Santana et al., 2012; Alexandre et al., 2015). Indeed, differences in body composition and in intermediary metabolism can impact on reproductive traits (Shaffer et al., 2011) and it has been observed before that feed efficient bulls present features of delayed sexual maturity, i.e., decreased progressive motility of the sperm and higher abundance of tail abnormalities (Fontoura et al., 2016; Montanholi et al., 2016). Moreover, high FE heifers presented lower fat deposition and later sexual maturity which results in calving later in the calving season than their low FE counterparts (Shaffer et al., 2011; Randel and Welsh, 2013). LFE animals also exhibit down-regulation of *AMH* and the decrease of this hormone in serum is an excellent marker of Sertoli cells pubertal development (Rey et al., 1993).

Concerning the differences in lipid metabolism in divergent FE phenotypes, FGF21, a hormone up-regulated in liver of HFE animals, is associated in humans to decrease in body weight, blood triglycerides and LDL-cholesterol, with improvement in insulin sensitivity (Cheung and Deng, 2014). It is an hepatokine released to the bloodstream and an important regulator of lipid and glucose metabolism (Giralt et al., 2015). When we performed an enrichment analysis of co-expressed genes with *FGF21*, we indeed found terms related to plasma lipoprotein particle remodeling, regulation of lipoprotein oxidation and cholesterol efflux mostly due to *FGF21* co-expression with the apolipoproteins APOA4, APOC3 and APOM. In the same context, pro-melanin-concentrating hormone (PMCH) encodes three neuropeptides: neuropeptide-glycine-glutamic acid, neuropeptide-glutamic acid-isoleucine and melanin-concentrating hormone (MCH), the last one being the most extensively studied (Helgeson and Schmutz, 2008). MCH up-regulation has been related to obesity and insulin resistance, as well as increased appetite and reduced metabolism in murine models (Ludwig et al., 2001; Ito et al., 2003). The *PMCH* gene is up-regulated in LFE animals and harbors SNPs found to be associated with higher carcass fat levels and marbling score (Helgeson and Schmutz, 2008; Walter et al., 2014).

In this work, we were able to identify several biological processes known to be related to feed efficiency, which together with the validation of the main transcription factors of the network, demonstrate the quality of the data and the robustness of the analyses, giving us the confidence to identify candidate genes as regulators or biomarkers of superior animals for this trait. The regulatory genes *NR2F6* and *TGFB1* play central roles in liver and muscle, respectively, by regulating genes related to inflammatory response and muscle development and growth, two main biological mechanisms associated to feed efficiency. Likewise, hormones and other proteins secreted in plasma as oxytocin, adrenomedulin, TSH, somatostatin, follistatin and AMH are interesting molecules to be explored as potential biomarkers of feed efficiency.

## Materials and Methods

### Phenotypic Data and Biological Sample Collection

All animal protocols were approved by the Institutional Animal Care and Use Committee of Faculty of Food Engineering and Animal Sciences, University of São Paulo (FZEA-USP – protocol number 14.1.636.74.1). All procedures to collect phenotypes and biological samples were carried out at FZEA-USP, Pirassununga, State of São Paulo, Brazil. Ninety-eight Nellore bulls (16 to 20 months old and 376 ± 29 kg BW) were evaluated in a feeding trial comprised of 21 days of adaptation to feedlot diet and place and a 70-day period of data collection. Total mixed ration was offered *ad libitum* and daily dry matter intake (DMI) was individually measured. Animals were weighed at the beginning, at the end and every 2 weeks during the experimental period. Feed efficiency was estimated by RFI which is the residual of the linear regression that estimates DMI based on average daily gain and mid-test metabolic body weight (Koch et al., 1963). 40 animals selected either as high feed efficiency (HFE) or low feed efficiency (LFE) groups were slaughtered on 2 days with a 6-day interval. Adrenal gland (longitudinal section), hypothalamus, liver (lateral portion of the left lobe), skeletal muscle (medial portion of *Longissimus lumborum*, close to 12th rib) and pituitary samples were collected from each animal, rapidly frozen in liquid nitrogen and stored at –80°C. Further information about management and phenotypic measures of the animals used in this study can be found in Alexandre et al. (2015).

### RNAseq Data Generation

Samples of nine animals from each feed efficiency group (high and low) were selected for RNAseq using RFI measure. For hypothalamus and pituitary, the nitrogen frozen tissue was macerated with crucibles and pistils to ensure all portions of the tissue were represented, and stored in aliquots at –80°C. Then, RNA was extracted using AllPrep DNA/RNA/Protein Mini kit (QIAGEN, Crawley, United Kingdom). For liver, muscle and adrenal gland, a cut was made in the frozen tissue and the RNA was extracted using RNeasy Mini Kit (QIAGEN, Crawley, United Kingdom). RNA quality and quantity were assessed using automated capillary gel electrophoresis on a Bioanalyzer 2100 with RNA 6000 Nano Labchips according to the manufacturer’s instructions (Agilent Technologies Ireland, Dublin, Ireland). Samples that presented an RNA integrity number (RIN) of less than 8.0 were discarded.

RNA libraries were constructed using the TruSeq^TM^ Stranded mRNA LT Sample Prep Protocol and sequenced on Illumina HiSeq 2500 equipment in a HiSeq Flow Cell v4 using the HiSeq SBS Kit V4 (2×100 pb). Liver, pituitary and hypothalamus were sequenced on the same run, each one in a different lane. Muscle and adrenal gland were sequenced in a second run, in different lanes.

### Gene Expression Estimation

The quality of the sequencing was evaluated using the software FastQC Version 3^1^. Sequence alignment against the bovine reference genome (UMD3.1) was performed using STAR Version 2.2.1 (Dobin et al., 2013), according to the standard parameters and including the annotation file (Ensembl release 89) and secondary alignments, duplicated reads and reads failing vendor quality checks were removed using Samtools Version 1.9 (Li et al., 2009). Then, HTseq Version 0.6.0 (Anders et al., 2014) was used to generate gene read counts and expression values were estimated by fragments per kilobase of gene per million mapped reads (FPKM). Genes with average value lower than 0.2 FPKM across all samples and tissues were discarded.

Gene expression normalization was performed using the following mixed effect model (Reverter et al., 2005):

$$\begin{array}{c} Y_{ijkl}=\mu +L_{i}+G_{j}+GT_{jk}+GP_{jl}+e_{ijkl} \end{array}$$

where, the log2-transformed FPKM value for *i*-th library (86 levels), *j*-th gene (17,354 levels), *k*-th tissue (5 levels), l-th RFI phenotype (2 levels), corresponding to *Y_ijkl_*, was modeled as a function of the fixed effect of library (*L_i_*) and the random effects of gene (*G_j_*), gene by tissue (*GT_jk_*) and gene by RFI phenotype (*GP_jl_*). Random residual (*e_ijkl_*) was assumed to be independent and identically distributed. Variance component estimates and solutions to the model were obtained using VCE6 (Groeneveld et al., 2010). Normalized mean expression (NME) values for each gene were defined as the linear combination of the solutions for random effects.

The mixed model used to normalize the expression data explained 96% of the variation in gene expression, of which the largest proportion (0.30) was due to tissue-specificity. Contrariwise, differences between HFE and LFE represented no variation (0.27 × 10^-11^). For that reason, normalized mean expression (NME) was only used to identify tissue specific genes and the raw FPKM values were used for differential expression and co-expression analysis.

### Gene Selection for Network Construction

In order to select a set of relevant genes for network analysis, we defined five categories based on the following inclusion criteria:

#### Differential Expression (DE)

The mean expression value of each gene, for each group (HFE and LFE) and each tissue was calculated and then the expression of LFE group was subtracted from the expression in HFE group. Next, genes were ranked according to their mean expression in all samples for each tissue and divided into five bins. Genes were considered differentially expressed when the difference between the expression in HFE and LFE groups were greater than 3.1 or smaller than – 3.1 standard deviation from the mean in each bin, corresponding to a *t*-test *P* < 0.001 (Weber et al., 2016).

#### Harboring SNPs

Genes harboring SNPs associated with feed efficiency, mainly indicated by GWAS, were identified using the PubMed database^2^ and the AnimalQTL database – Release 35^3^ and only bovine data were considered regardless of breed.

#### Tissue Specific (TS)

A gene was considered as tissue specific when the average NME in that tissue was greater than one standard deviation from the mean of all genes and the average NME in all the other four tissues was smaller than zero.

#### Secreted

The human secretome database^4^ (Uhlén et al., 2005; Uhlen et al., 2015) was used to select genes encoding proteins secreted in plasma by any of the analyzed tissues (adrenal gland, hypothalamus, liver, muscle and pituitary).

#### Key Regulators

In order to identify key regulatory genes to be included in the co-expression network, a list of genes were obtained from the Animal Transcription Factor Database 2.0^5^ (Zhang et al., 2015) and it was compared to a set of potential target genes in each tissue, composed of the categories: TS, DE, harboring SNPs and secreted. The analysis was based on regulatory impact factor metrics (Reverter et al., 2010), which comprises a set of two metrics designed to assign scores to regulator genes consistently differentially co-expressed with target genes and to those with the most altered ability to predict the abundance of target genes. Those scores deviating ± 1.96 standard deviation from the mean (corresponding to *P* < 0.05) were considered significant. Genes presenting mean expression value less than the mean of all genes expressed were not considered in this analysis.

Some of the genes selected by the categories above were represented by more than one Ensembl ID. Those duplications were removed for further analysis, keeping only the expression value of the most meaningful Ensembl ID. Additionally, genes with mean expression across the samples equal to zero were also removed from further analysis.

### Co-expression Network Analysis

For gene network inference, genes selected using the five categories described previously were used as nodes and significant connections (edges) between them were identified using the Partial Correlation and Information Theory (PCIT) algorithm (Reverter and Chan, 2008), considering all animals and all tissues. PCIT determinates the significance of the correlation between two nodes after accounting for all the other nodes in the network. Connections between gene nodes were accepted when the partial correlation was greater than two standard deviations from the mean (*P* < 0.01). The output of PCIT was visualized on Cytoscape Version 3.6.1 (Shannon et al., 2003).

### Network Validation Through Transcription Factor Biding Motifs Analysis

Using the regulatory impact factor metric (RIF) we prioritized key regulator genes from gene expression data and predicted target genes based on co-expression network. In order to assess whether those target genes were enriched for motifs associated with the top most connected regulators in the network with a DNA binding domain (transcription factors – TF), we performed motif discovery analysis in the set of co-expressed target genes (first neighbors of the TF) using the i-cistarget method (Herrmann et al., 2012) and i-Regulon v1.3, a Cytoscape plug-in (Janky et al., 2014). These tools use humans (hg19) as the reference species, therefore only genes with human orthology are assessed. Then, to validate the binding of the identified genomic regions by the TFs, we performed a region enrichment analysis across experimentally available TF bound regions from ChiP-seq in cell lines from the ENCODE consortium (1,394 TF binding site tracks, Encode Project Consortium, 2012; Sloan et al., 2016). Briefly, the tools evaluate whether there is an over-representation of motifs in the set of co-expressed genes and across evolution. We examined 10 kb upstream of the gene transcription start site and their conservation in 7 vertebrate species, including cow. Thus, the tools provide over-represented motifs across evolution, allowing us to predict regulatory interactions TF to target gene in cow. In our analysis, we performed motif discovery using i-Regulon v1.3 (Janky et al., 2014) and i-cistarget database 3 (Herrmann et al., 2012), that is using their 9713 motif collection. Both methods result in highly similar enrichments. Whereas i-Regulon is a user-friendly method to deliver a regulatory network, i-cistarget also yields the genomic position of the TF binding in the genome. Both i-Regulon and i-cistarget can be used to validate the TF binding on predicted genomic regions in the human genome. The tool contains a collection of TF ChIP-seq data in cell lines mostly from the ENCODE consortium (1,394 TF binding tracks), 2003 Histone modifications from the ENCODE consortium and Epigenomics roadmap and 908 Histone modification and open-chromatin. The tool allows to perform an enrichment of the different human tracks at the region level.

Finally, we converted identified enhancer regions into cow coordinates and searched for regions of open-chromatin using data from publicly available studies in cow tissues. Namely, cow liver promoters and enhancer from Villar et al. (2015) (Array Express Accession number E-MTAB-2633) and skeletal muscle cow promoters from Zhao et al. (2015) (GSE61936). For *MYOD1* and *SMAD3* binding in myotubes and pro-B cells, data from Mullen et al. (2011) was used (GEO: GSE21621); and for *MYOD1* binding in primary myotubes, data from Cao et al. (2010) (GEO: GSE20059) was used.

### Differential Connectivity

In order to explore differentially connected genes between HFE and LFE, two networks were created, one for each condition, using the same methodology described before. Then, the number of connections of each gene in each condition was computed and scaled so that connectivity varied from 0 to 1, making it possible to compare the same gene in the two networks. The connectivity in LFE group was subtracted from the connectivity in HFE group and results deviating ± 1.96 standard deviation from the mean were considered significant (*P* < 0.05).

### Functional Enrichment

Functional enrichment analysis was performed on the online platform GOrilla (Gene Ontology enRIchment anaLysis and visuaLizAtion tool^6^), using all genes that passed FPKM filter as background, hypergeometric test and multiple test correction (FDR – false discovery rate). The human database was used to take advantage of a more comprehensive knowledgebase regarding gene functions. GO terms were considered significant when Padj < 0.05. For genes in co-expression networks, visualized using Cytoscape (Shannon et al., 2003), the functional enrichment was performed with BiNGO plug-in (Maere et al., 2005) using the same background genes and statistical test.

## Data Availability

Datasets supporting the results of this article are public available in the European Nucleotide Archive (ENA) as part of FAANG consortium under de study ID PRJEB27337 and can be accessed following the link https://www.ebi.ac.uk/ena/data/view/PRJEB27337. Moreover, any additional information as well as the scripts used to perform the analysis are available upon request, please email pamela.alexandre@usp.br. This manuscript has been published as a preprint in bioRxiv, doi: https://doi.org/10.1101/360396.

## Author Contributions

PA performed formal analysis, investigation and visualization of all presented data and wrote the original draft. HF was the overall project leader responsible for conceptualization, project administration and supervision of PA. AR performed bioinformatics analysis and investigation and supervised PA. MN-S performed bioinformatics analysis and investigation. JF was responsible for funding acquisition and project administration. LP-N provided computing resources and conceptualization. All authors contributed to reviewing and editing of this manuscript’s final version.

## Conflict of Interest Statement

The authors declare that the research was conducted in the absence of any commercial or financial relationships that could be construed as a potential conflict of interest.

## Footnotes

1 http://www.bioinformatics.babraham.ac.uk/projects/fastqc/

2 www.ncbi.nlm.nih.gov/pubmed/

3 www.animalgenome.org/cgi-bin/QTLdb/index

4 www.proteinatlas.org/humanproteome/secretome

5 http://bioinfo.life.hust.edu.cn/AnimalTFDB/#!/

6 http://cbl-gorilla.cs.technion.ac.il/
