# Supplementary material for: Systems Biology Reveals NR2F6 and TGFB1 as Key Regulators of Feed Efficiency in Beef Cattle
Source: Front Genet. 2019 Mar 22;10:230. doi: 10.3389/fgene.2019.00230 (PMC6439317; doi:10.3389/fgene.2019.00230)
Supplement: Supplementary file 12 [file Data_Sheet_1.PDF]

i-cisTarget

An integrative genomics method for the prediction of regulatory features and cis-regulatory modules.

Parameters and statistics for NR2F6 co-expressed genes

Number of features	9713
Number of enriched features (NES > 3.0)	79
Total number of ranked regions	220330
Type of input query	hgnc_symbol
Fraction of mapped input IDs	0.884
Number of **i-cisTarget regions in input set**	2822
Minimum fraction of overlap	0.4
Normalized enrichment score (NES) threshold	3.0
AUC threshold (fraction / # of ranked regions)	0.005 (1102)
Recovery curve threshold (# of regions)	20000

AUC distribution

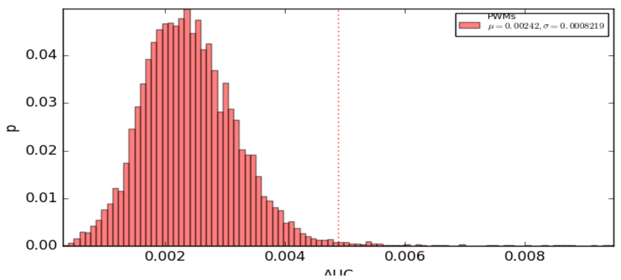

Recovery of best feature

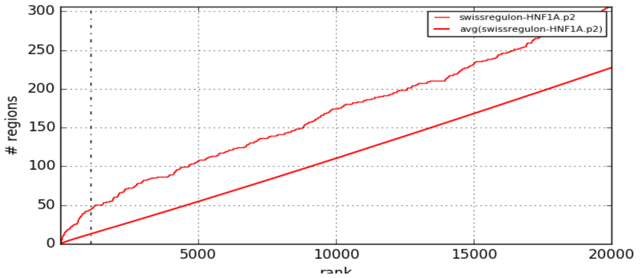

Results for NR2F6 co-expressed genes

Select features in the table below, select an operation and proceed.

1. Use candidate target regions as **filter** and use as input for i-cisTarget again.
2. **Scan** candidate target regions of selected features for multiple homotypic CRMs.
3. **Create SIF file** for the selected features.

This report is also available as an **archive**.

#	Feature	NES	Logo	Recovery Curve	Candidate targets	All regions in top 20000	Database
1	swissregulon-HNF1A.p2<br>Description: HNF1A<br>Possible TFs: HNF1A	*8.58959*	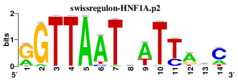	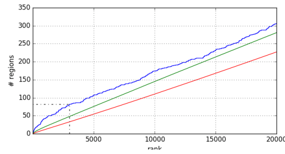	link	link	PWMs
2	transfac_pro-M02162<br>Description: V$HNF1A_01<br>Possible TFs: HNF1A	*8.40766*	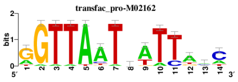	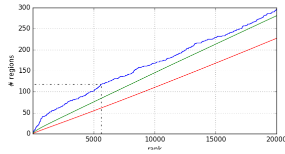	link	link	PWMs
3	transfac_public-M00132<br>Description: V$HNF1_01<br>Possible TFs: HNF1A	*8.17956*	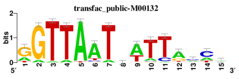	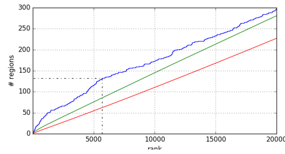	link	link	PWMs
4	taipale-NRTTAATNATTAACN-HNF1B-full<br>Description: NRTTAATNATTAACN-HNF1B-full	*7.79028*	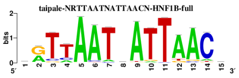	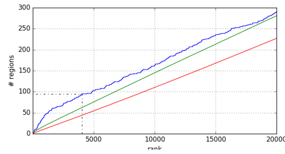	link	link	PWMs

#	Feature	NES	Logo	Recovery Curve	Candidate targets	All regions in top 20000	Database
5	taipale-NRTTAATNATTAACN-HNF1A-full<br>Description: NRTTAATNATTAACN-HNF1A-full	7.61109	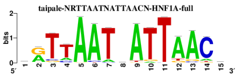	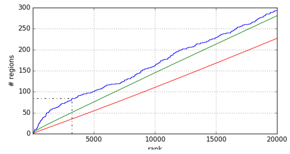	link	link	PWMs
6	taipale-GTTAATNATTAAY-HNF1B-full<br>Description: GTTAATNATTAAY-HNF1B-full	7.49919	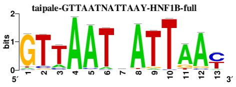	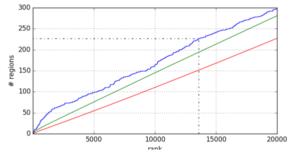	link	link	PWMs
7	transfac_pro-M01031<br>Description: V$HNF4_Q6_01<br>Possible TFs: HNF4A	7.40608	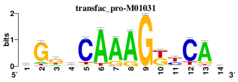	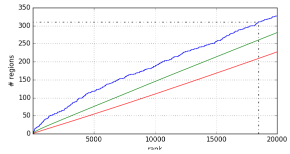	link	link	PWMs
8	homer-M00086<br>Description: Hnf1(Homeobox)/Liver-Foxa2-Chip-Seq/Homer<br>Possible TFs: HNF1B, HNF1A	7.20615	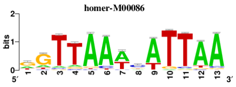	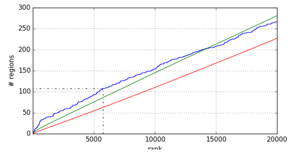	link	link	PWMs
9	transfac_pro-M02220<br>Description: V$HNF4A_03<br>Possible TFs: HNF4A	7.16351	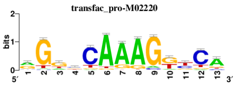	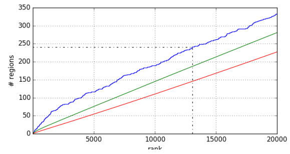	link	link	PWMs
10	jaspar-PF0084.1<br>Description: RGTTAMWNATT	6.84230	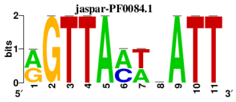	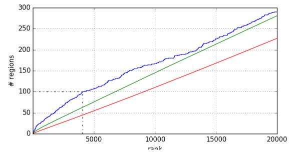	link	link	PWMs
11	transfac_pro-M03826<br>Description: V$HNF1B_Q6<br>Possible TFs: HNF1B	6.49918	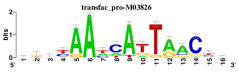	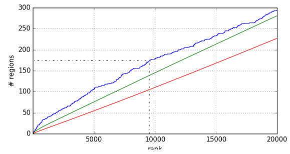	link	link	PWMs
12	factorbook-HNF4<br>Description: HNF4<br>Possible TFs: HNF4G, HNF4A	6.41272	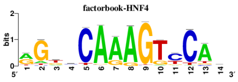	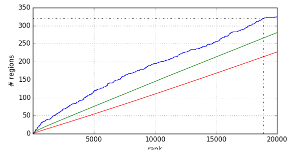	link	link	PWMs
13	tfdimers-MD00041<br>Description: Hmbox1_CRX	6.38298	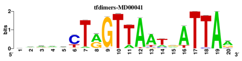	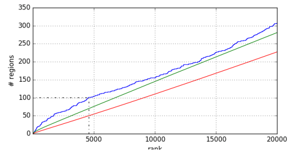	link	link	PWMs
14	transfac_pro-M00764<br>Description: V$HNF4_DR1_Q3<br>Possible TFs: HNF4G, HNF4A	6.19088	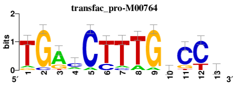	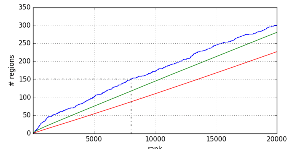	link	link	PWMs
15	transfac_pro-M02016<br>Description: V$HNF4A_Q6_01<br>Possible TFs: HNF4A	6.18893	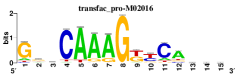	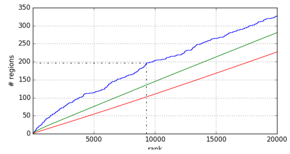	link	link	PWMs

#	Feature	NES	Logo	Recovery Curve	Candidate targets	All regions in top 20000	Database
16	transfac_pro-M03828<br>Description: V$HNF4_Q6_04<br>Possible TFs: HNF4A	*6.08798*	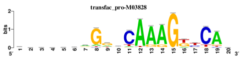	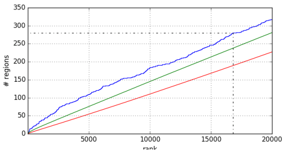	link	link	PWMs
17	transfac_pro-M01011<br>Description: V$HNF1_Q6_01<br>Possible TFs: HNF1B, HNF1A	*5.55237*	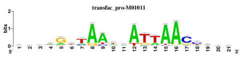	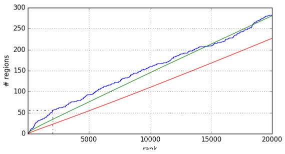	link	link	PWMs
18	transfac_pro-M02013<br>Description: V$HNF1A_Q5<br>Possible TFs: HNF1A	*5.55003*	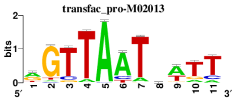	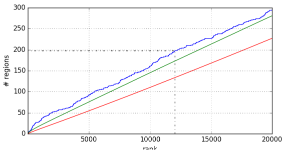	link	link	PWMs
19	transfac_pro-M00967<br>Description: V$HNF4_Q6<br>Possible TFs: NR2F1, HNF4G, HNF4A, NR2F2	*5.53086*	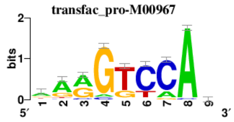	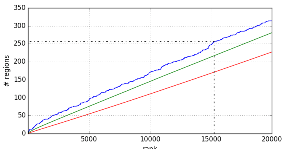	link	link	PWMs
20	transfac_public-M00134<br>Description: V$HNF4_01<br>Possible TFs: HNF4A	*5.22686*	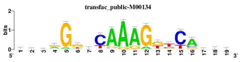	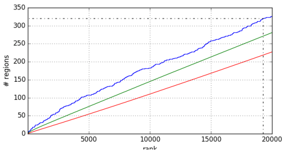	link	link	PWMs
21	swissregulon-HNF4A_NR2F1-2.p2<br>Description: NR2F1, NR2F2, HNF4A	*5.06567*	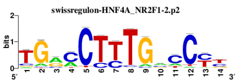	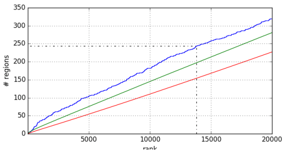	link	link	PWMs
22	transfac_pro-M00790<br>Description: V$HNF1_Q6<br>Possible TFs: HNF1B, HNF1A	*4.94008*	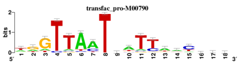	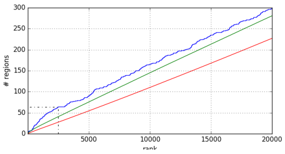	link	link	PWMs
23	transfac_pro-M02868<br>Description: V$HNF4A_02<br>Possible TFs: HNF4A	*4.78398*	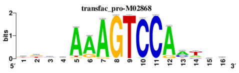	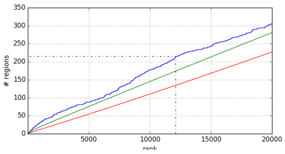	link	link	PWMs
24	transfac_pro-M01107<br>Description: V$RUSH1A_02<br>Possible TFs: HLTF	*4.70651*	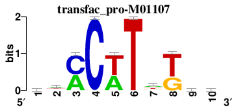	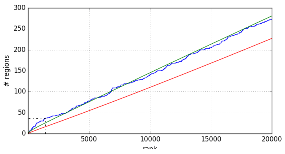	link	link	PWMs
25	transfac_pro-M00912<br>Description: V$CEBP_Q2_01<br>Possible TFs: CEBPB, CEBPA, CEBPG, CEBPE, CEBPD	*4.62357*	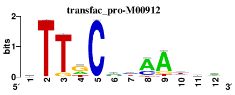	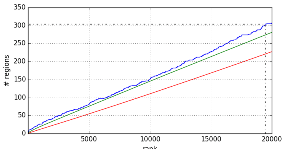	link	link	PWMs
26	transfac_public-M00158<br>Description: V$COUP_01<br>Possible TFs: NR2F1, HNF4A	*4.60166*	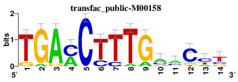	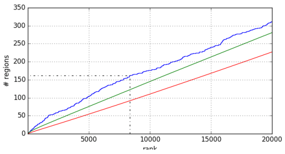	link	link	PWMs

#	Feature	NES	Logo	Recovery Curve	Candidate targets	All regions in top 20000	Database
27	transfac_pro-M00770<br>Description: V$CEBP_Q3<br>Possible TFs: CEBPB, CEBPA, CEBPG, CEBPE, CEBPD	4.57779	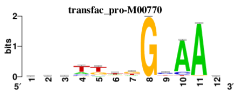	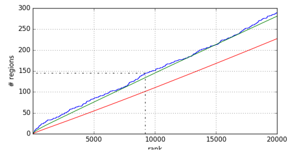	link	link	PWMs
28	transfac_pro-M00638<br>Description: V$HNF4ALPHA_Q6<br>Possible TFs: HNF4A	4.43381	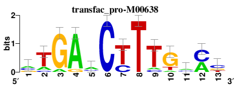	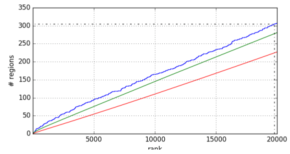	link	link	PWMs
29	tfdimers-MD00358<br>Description: HNF3A_RUSH-1alpha	4.35439	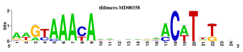	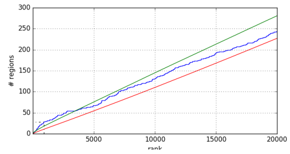	link	link	PWMs
30	encode-UW.Motif.0020<br>Description: UW.Motif.0020	4.34344	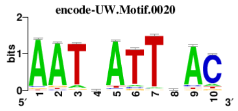	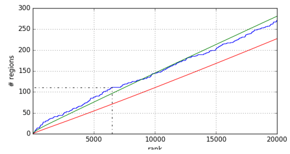	link	link	PWMs
31	homer-M00087<br>Description: HNF4a(NR/DR1)/HepG2-HNF4a-ChIP-Seq/Homer<br>Possible TFs: HNF4A	4.33757	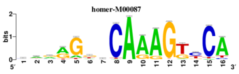	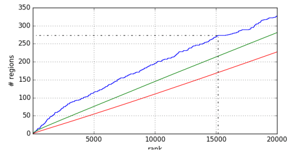	link	link	PWMs
32	taipale-RRGGTCAAAGTCCRNN-HNF4A-DBD<br>Description: RRGGTCAAAGTCCRNN-HNF4A-DBD	4.15760	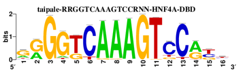	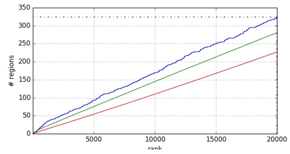	link	link	PWMs
33	transfac_pro-M02266<br>Description: V$HNF1B_04<br>Possible TFs: HNF1B	4.02849	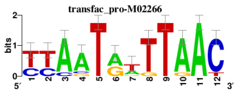	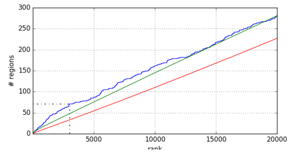	link	link	PWMs
34	transfac_pro-M00762<br>Description: V$DR1_Q3<br>Possible TFs: NR2F1, HNF4G, HNF4A, NR2F2	3.92324	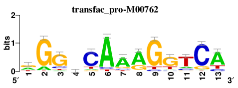	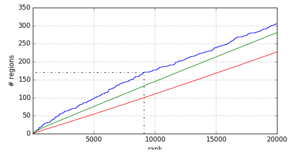	link	link	PWMs
35	tfdimers-MD00020<br>Description: MYB_Pax-4	3.90212	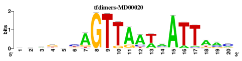	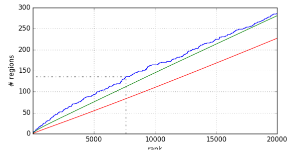	link	link	PWMs
36	transfac_public-M00116<br>Description: V$CEBPA_01<br>Possible TFs: CEBPA	3.87903	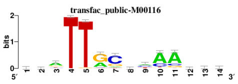	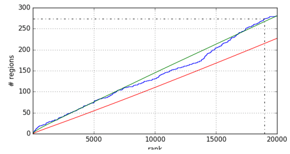	link	link	PWMs
37	taipale-NRGTCCAAAGTCCANY-HNF4A-full<br>Description: NRGTCCAAAGTCCANY-HNF4A-full	3.87747	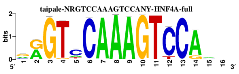	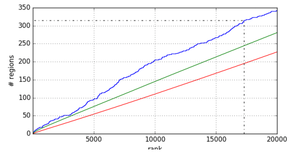	link	link	PWMs

#	Feature	NES	Logo	Recovery Curve	Candidate targets	All regions in top 20000	Database
38	homer-M00047<br>Description: Erra(NR)/HepG2-Erra-ChIP-Seq/Homer<br>Possible TFs: ESRRA	*3.87356*	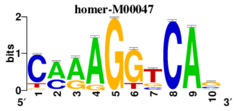	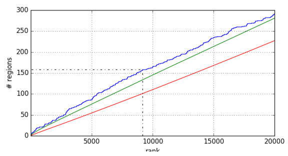	**link**	**link**	PWMs
39	transfac_pro-M01866<br>Description: V$CEBPA_Q6<br>Possible TFs: CEBPA	*3.85791*	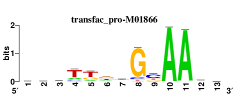	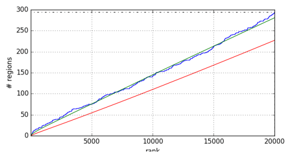	**link**	**link**	PWMs
40	transfac_public-M00206<br>Description: V$HNF1_C<br>Possible TFs: HNF1A	*3.85438*	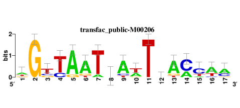	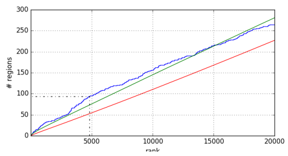	**link**	**link**	PWMs
41	taipale-RRGTCCAAAGGTCAA-HNF4A-full<br>Description: RRGTCCAAAGGTCAA-HNF4A-full	*3.75266*	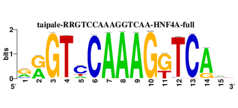	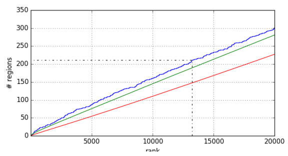	**link**	**link**	PWMs
42	homer-M00186<br>Description: Stat3+il23(Stat)/CD4-Stat3-ChIP-Seq/Homer<br>Possible TFs: STAT3	*3.74562*	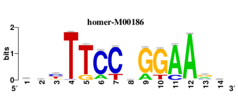	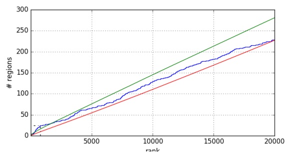	**link**	**link**	PWMs
43	transfac_public-M00411<br>Description: V$HNF4_01_B<br>Possible TFs: HNF4A	*3.74210*	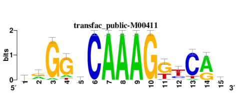	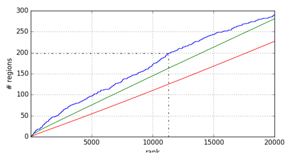	**link**	**link**	PWMs
44	homer-M00001<br>Description: AARE(HLH)/mES-cMyc-ChIP-Seq/Homer	*3.71980*	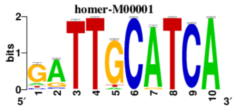	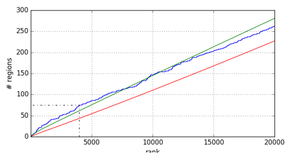	**link**	**link**	PWMs
45	homer-M00170<br>Description: RXR(NR/DR1)/3T3L1-RXR-ChIP-Seq/Homer<br>Possible TFs: RXRA	*3.63490*	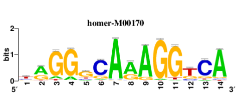	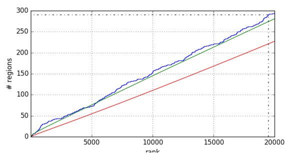	**link**	**link**	PWMs
46	encode-UW.Motif.0360<br>Description: UW.Motif.0360	*3.62277*	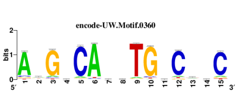	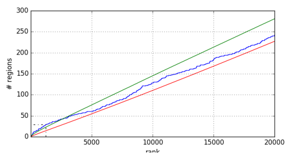	**link**	**link**	PWMs
47	transfac_pro-M03543<br>Description: V$COUPTF2_Q6	*3.61612*	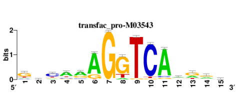	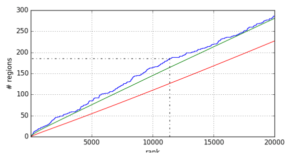	**link**	**link**	PWMs
48	tfdimers-MD00042<br>Description: CRX_Oct-1	*3.60908*	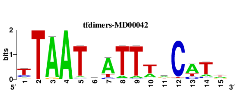	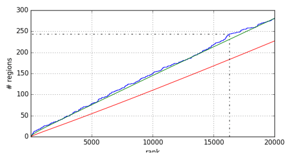	**link**	**link**	PWMs

#	Feature	NES	Logo	Recovery Curve	Candidate targets	All regions in top 20000	Database
49	transfac_public-M00223<br>Description: V$STAT_01<br>Possible TFs: STAT6, STAT4, STAT3, STAT2, STAT1, STAT5B, STAT5A	*3.60164*	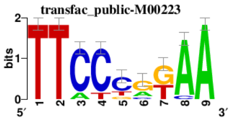	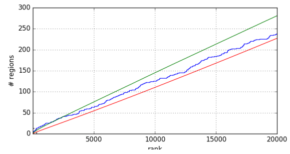	link	link	PWMs
50	tfdimers-MD00124<br>Description: HNF4, COUP_IPF1	*3.59929*	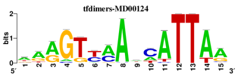	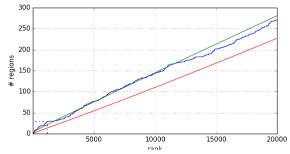	link	link	PWMs
51	swissregulon-STAT2-4-6.p2<br>Description: STAT2, STAT4, STAT6	*3.58443*	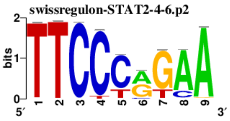	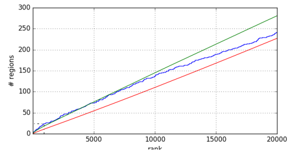	link	link	PWMs
52	taipale-RRGGTCAAAGGTCA-HNF4A-full<br>Description: RRGGTCAAAGGTCA-HNF4A-full	*3.57308*	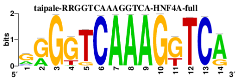	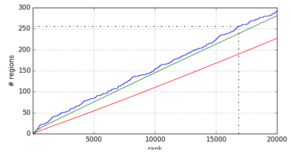	link	link	PWMs
53	tfdimers-MD00076<br>Description: HNF1_PPARG	*3.56800*	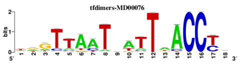	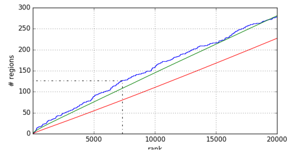	link	link	PWMs
54	tfdimers-MD00289<br>Description: HNF1_IRX4	*3.51870*	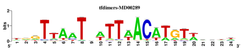	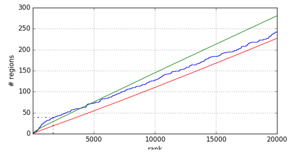	link	link	PWMs
55	taipale-RRGGTCAAAGTCCRNN-Hnf4a-DBD<br>Description: RRGGTCAAAGTCCRNN-Hnf4a-DBD	*3.47136*	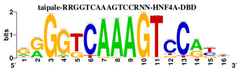	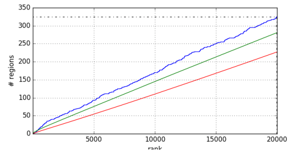	link	link	PWMs
56	tfdimers-MD00272<br>Description: ISL1_Tst-1	*3.45336*	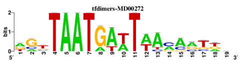	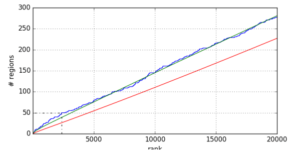	link	link	PWMs
57	factorbook-RXRA<br>Description: RXRA<br>Possible TFs: RXRA	*3.44319*	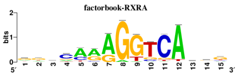	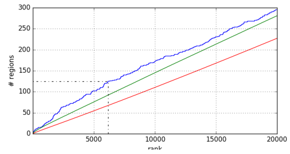	link	link	PWMs
58	homer-M00154<br>Description: PR(NR)/T47D-PR-ChIP-Seq(GSE31130)/Homer<br>Possible TFs: PGR	*3.42089*	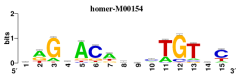	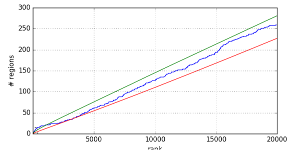	link	link	PWMs
59	flyfactorsurvey-CG15601_SANGER_5_FBgn0030673<br>Description: FBgn0030673(CG15601)	*3.38802*	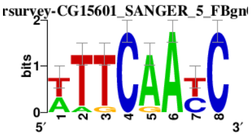	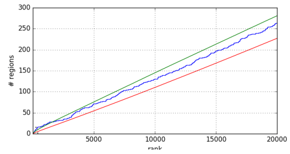	link	link	PWMs

#	Feature	NES	Logo	Recovery Curve	Candidate targets	All regions in top 20000	Database
60	encode-UW.Motif.0036<br>Description: UW.Motif.0036	3.37042	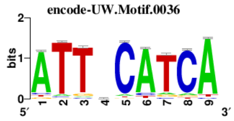	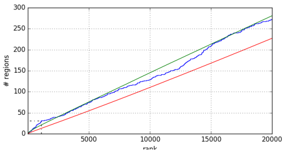	link	link	PWMs
61	tfdimers-MD00248<br>Description: AML1a_HNF1	3.26635	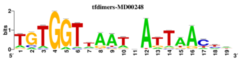	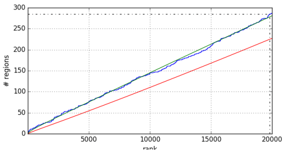	link	link	PWMs
62	hdpi-XG<br>Description: XG<br>Possible TFs: XG	3.26244	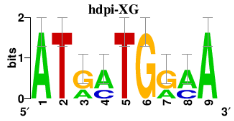	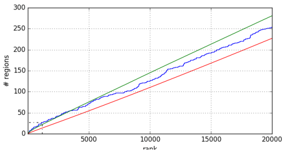	link	link	PWMs
63	transfac_public-M00457<br>Description: V$STAT5A_01<br>Possible TFs: STAT5A	3.26087	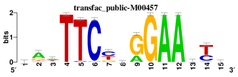	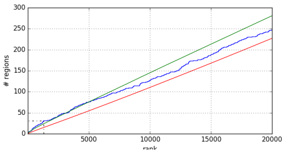	link	link	PWMs
64	transfac_pro-M01595<br>Description: V$STAT3_03<br>Possible TFs: STAT3	3.24913	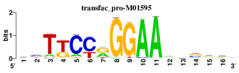	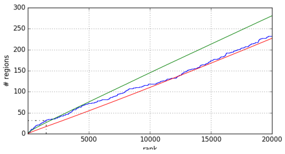	link	link	PWMs
65	factorbook-CEBPB<br>Description: CEBPB<br>Possible TFs: CEBPB	3.21549	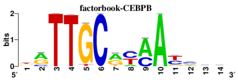	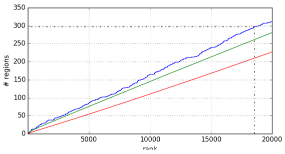	link	link	PWMs
66	taipale-NRNWAAYRTTKNYN-FOXD2-DBD<br>Description: NRNWAAYRTTKNYN-FOXD2-DBD	3.19514	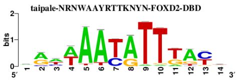	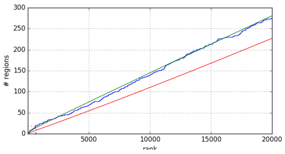	link	link	PWMs
67	jaspar-MA0066.1<br>Description: PPARG<br>Possible TFs: PPARG	3.16815	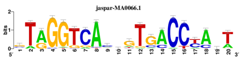	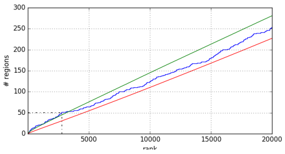	link	link	PWMs
68	taipale-TGAATRTKCAGTCA-SOX10-full<br>Description: TGAATRTKCAGTCA-SOX10-full	3.15093	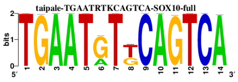	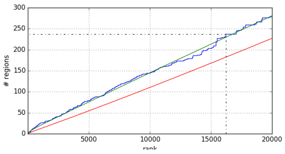	link	link	PWMs
69	transfac_pro-M03147<br>Description: I$KNI_01	3.12980	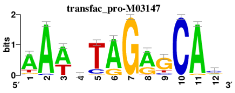	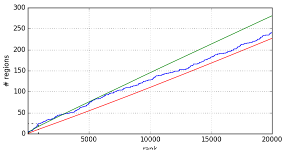	link	link	PWMs
70	homer-M00542<br>Description: kni/dmmpmm(Noyes)/fly<br>Possible TFs: RORC, RORB, RORA	3.12785	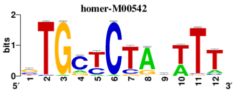	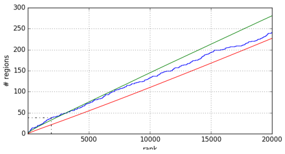	link	link	PWMs

#	Feature	NES	Logo	Recovery Curve	Candidate targets	All regions in top 20000	Database
71	encode-UW.Motif.0449<br>Description: UW.Motif.0449	*3.12433*	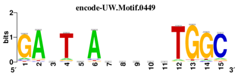	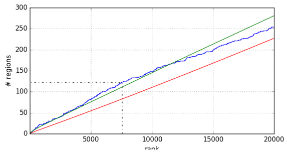	**link**	**link**	PWMs
72	transfac_public-M00105<br>Description: V$CDPCR3_01<br>Possible TFs: CUX1	*3.10398*	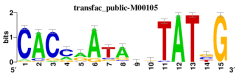	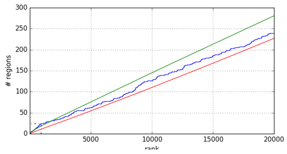	**link**	**link**	PWMs
73	transfac_pro-M00806<br>Description: V$NF1_Q6_01<br>Possible TFs: NFIC, NFIA	*3.08677*	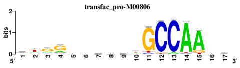	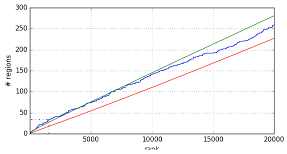	**link**	**link**	PWMs
74	taipale-RRGGTCAAAGTCCRNN-HNF4A-full<br>Description: RRGGTCAAAGTCCRNN-HNF4A-full	*3.08325*	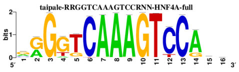	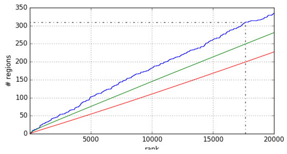	**link**	**link**	PWMs
75	tfdimers-MD00247<br>Description: HOXA10_CRX	*3.06721*	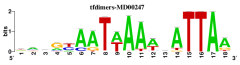	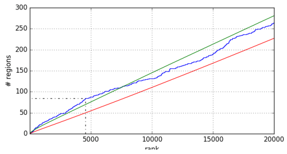	**link**	**link**	PWMs
76	homer-M01498<br>Description: MAC1/Literature(Harbison)/Yeast	*3.05077*	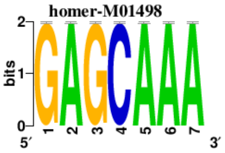	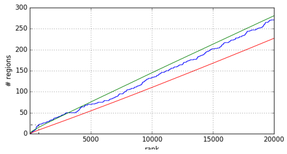	**link**	**link**	PWMs
77	taipale-NNATGAYGCAATN-ATF4-DBD<br>Description: NNATGAYGCAATN-ATF4-DBD	*3.04099*	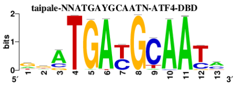	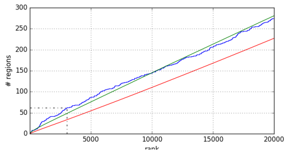	**link**	**link**	PWMs
78	jaspar-MA0137.2<br>Description: STAT1	*3.00930*	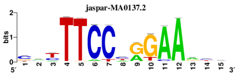	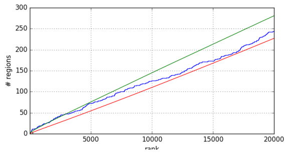	**link**	**link**	PWMs
79	homer-M00189<br>Description: STAT5(Stat)/mCD4+-Stat5a|b-ChIP-Seq/Homer	*3.00695*	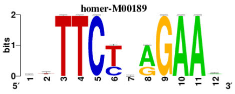	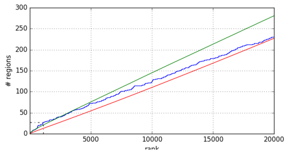	**link**	**link**	PWMs

© 2015 **KULeuven** | Authors: Gert Hulselmans & Hana Imrichova | Web design (CSS) by **Andreas Viklund**.
