# Supplementary material for: Systems Biology Reveals NR2F6 and TGFB1 as Key Regulators of Feed Efficiency in Beef Cattle
Source: Front Genet. 2019 Mar 22;10:230. doi: 10.3389/fgene.2019.00230 (PMC6439317; doi:10.3389/fgene.2019.00230)
Supplement: Supplementary file 13 [file Data_Sheet_2.PDF]

# i-cisTarget

An integrative genomics method for the prediction of regulatory features and cis-regulatory modules.

## Parameters and statistics for TGB1 co-expressed genes

| | |
|---|---|
| Number of features | 9713 |
| Number of enriched features (NES > 3.0) | 107 |
| Total number of ranked regions | 220330 |
| Type of input query | hgnc_symbol |
| Fraction of mapped input IDs | 0.896 |
| Number of i-cisTarget regions in input set | 2195 |
| Minimum fraction of overlap | 0.4 |
| Normalized enrichment score (NES) threshold | 3.0 |
| AUC threshold (fraction / # of ranked regions) | 0.005 (1102) |
| Recovery curve threshold (# of regions) | 20000 |

**AUC distribution** AUC distribution image

**Recovery of best feature** RCC of best feature image

## Results for TGB1 co-expressed genes

Select features in the table below, select an operation and proceed.

1. Use candidate target regions as **filter** and use as input for i-cisTarget again.
2. **Scan** candidate target regions of selected features for multiple homotypic ▼ CRMs.
3. **Create SIF file** for the selected features.

This report is also available as an archive.

| # | Feature | NES | Logo | Recovery Curve | Candidate targets | All regions in top 20000 | Databa |
|---|---|---|---|---|---|---|---|
| 1 | transfac_public-M00231 Description: V$MEF2_02 Possible TFs: MEF2A | *10.91485* | transfac_public-M00231 logo | RCC of transfac_public-M00231 | link | link | PWMs |
| 2 | yetfasco-271 Description: YPL089C Possible TFs: MEF2C, MEF2B, MEF2A, MEF2BNB-MEF2B, MEF2D | *10.64097* | yetfasco-271 logo | RCC of yetfasco-271 | link | link | PWMs |
| 3 | jaspar-MA0052.1 Description: MEF2A Possible TFs: MEF2A | *9.83646* | jaspar-MA0052.1 logo | RCC of jaspar-MA0052.1 | link | link | PWMs |
| 4 | taipale-KCTAWAAATAGM-MEF2A-DBD Description: KCTAWAAATAGM-MEF2A-DBD | *9.64780* | taipale-KCTAWAAATAGM-MEF2A-DBD logo | RCC of taipale-KCTAWAAATAGM-MEF2A-DBD | link | link | PWMs |
| 5 | factorbook-MEF2 Description: MEF2 Possible TFs: MEF2C, MEF2A | *9.42673* | factorbook-MEF2 logo | RCC of factorbook-MEF2 | link | link | PWMs |
| 6 | swissregulon-MEF2_A-B-C-D_.p2 Description: MEF2A, MEF2C, MEF2D, MEF2BNB-MEF2B, MEF2B | *9.34297* | swissregulon-MEF2_A-B-C-D_.p2 logo | RCC of swissregulon-MEF2_A-B-C-D_.p2 | link | link | PWMs |
| 7 | yetfasco-419 Description: YPL089C Possible TFs: MEF2C, MEF2B, MEF2A, MEF2BNB-MEF2B, MEF2D | *9.33168* | yetfasco-419 logo | RCC of yetfasco-419 | link | link | PWMs |
| 8 | homer-M00110 Description: Mef2c(MADS)/GM12878-Mef2c-ChIP-Seq(GSE32465)/Homer Possible TFs: MEF2C | *9.19474* | homer-M00110 logo | RCC of homer-M00110 | link | link | PWMs |
| 9 | transfac_public-M00026 Description: V$RSRFC4_01 Possible TFs: MEF2A | *9.16852* | transfac_public-M00026 logo | RCC of transfac_public-M00026 | link | link | PWMs |
| 10 | taipale-NCTAWAAATAGM-MEF2D-DBD Description: NCTAWAAATAGM-MEF2D-DBD | *9.10770* | taipale-NCTAWAAATAGM-MEF2D-DBD logo | RCC of taipale-NCTAWAAATAGM-MEF2D-DBD | link | link | PWMs |
| 11 | transfac_pro-M00941 Description: V$MEF2_Q6_01 Possible TFs: MEF2C, MEF2A, MEF2D | *8.90338* | transfac_pro-M00941 logo | RCC of transfac_pro-M00941 | link | link | PWMs |
| 12 | jaspar-PF0028.1 Description: CTAWWWATA | *8.88408* | jaspar-PF0028.1 logo | RCC of jaspar-PF0028.1 | link | link | PWMs |
| 13 | transfac_pro-M02488 Description: F$RLM1_02 | *8.66156* | transfac_pro-M02488 logo | RCC of transfac_pro-M02488 | link | link | PWMs |

| # | Feature | NES | Logo | Recovery Curve | Candidate targets | All regions in top 20000 | Databa |
|---|---|---|---|---|---|---|---|
| 14 | homer-M00109<br>Description: Mef2a(MADS)/HL1-Mef2a.biotin-ChIP-Seq/Homer/<br>Possible TFs: MEF2A | 8.55193 | homer-M00109 logo | RCC of homer-M00109 | link | link | PWMs |
| 15 | transfac_public-M00232<br>Description: V$MEF2_03<br>Possible TFs: MEF2A | 8.45578 | transfac_public-M00232 logo | RCC of transfac_public-M00232 | link | link | PWMs |
| 16 | homer-M00298<br>Description: MF0008.1_MADS_class/Jaspar | 8.45433 | homer-M00298 logo | RCC of homer-M00298 | link | link | PWMs |
| 17 | jaspar-MA0001.1<br>Description: AGL3 | 8.29444 | jaspar-MA0001.1 logo | RCC of jaspar-MA0001.1 | link | link | PWMs |
| 18 | taipale-RCTAWAAATAGM-MEF2B-full<br>Description: RCTAWAAATAGM-MEF2B-full | 8.29444 | taipale-RCTAWAAATAGM-MEF2B-full logo | RCC of taipale-RCTAWAAATAGM-MEF2B-full | link | link | PWMs |
| 19 | jaspar-MF0008.1<br>Description: MADS_class | 8.28315 | jaspar-MF0008.1 logo | RCC of jaspar-MF0008.1 | link | link | PWMs |
| 20 | transfac_pro-M00407<br>Description: V$RSRFC4_Q2<br>Possible TFs: MEF2A | 8.16843 | transfac_pro-M00407 logo | RCC of transfac_pro-M00407 | link | link | PWMs |
| 21 | yetfasco-1428<br>Description: YPL089C<br>Possible TFs: MEF2C, MEF2B, MEF2A, MEF2BNB-MEF2B, MEF2D | 8.16224 | yetfasco-1428 logo | RCC of yetfasco-1428 | link | link | PWMs |
| 22 | stark-GNCTANWWATA<br>Description: GNCTANWWATA | 7.94955 | stark-GNCTANWWATA logo | RCC of stark-GNCTANWWATA | link | link | PWMs |
| 23 | transfac_pro-M01301<br>Description: V$MEF2_05<br>Possible TFs: MEF2A | 7.94154 | transfac_pro-M01301 logo | RCC of transfac_pro-M01301 | link | link | PWMs |
| 24 | yetfasco-1501<br>Description: YPL089C<br>Possible TFs: MEF2C, MEF2B, MEF2A, MEF2BNB-MEF2B, MEF2D | 7.94154 | yetfasco-1501 logo | RCC of yetfasco-1501 | link | link | PWMs |
| 25 | homer-M01523<br>Description: RLM1/Literature(Harbison)/Yeast | 7.80897 | homer-M01523 logo | RCC of homer-M01523 | link | link | PWMs |
| 26 | transfac_pro-M01809<br>Description: P$RIN_01 | 7.79185 | transfac_pro-M01809 logo | RCC of transfac_pro-M01809 | link | link | PWMs |
| 27 | stark-YTAWWWWTAR<br>Description: YTAWWWWTAR(Mef2)<br>Possible TFs: MEF2C, MEF2B, MEF2A, MEF2BNB-MEF2B, MEF2D | 7.25393 | stark-YTAWWWWTAR logo | RCC of stark-YTAWWWWTAR | link | link | PWMs |
| 28 | transfac_pro-M01007<br>Description: V$SRF_Q5_02<br>Possible TFs: SRF | 7.22917 | transfac_pro-M01007 logo | RCC of transfac_pro-M01007 | link | link | PWMs |
| 29 | homer-M00183<br>Description: CArG(MADS)/PUER-Srf-ChIP-Seq/Homer<br>Possible TFs: SRF | 7.05763 | homer-M00183 logo | RCC of homer-M00183 | link | link | PWMs |
| 30 | jaspar-PF0054.1<br>Description: TAAWWATAG | 7.04488 | jaspar-PF0054.1 logo | RCC of jaspar-PF0054.1 | link | link | PWMs |
| 31 | transfac_public-M00215<br>Description: V$SRF_C<br>Possible TFs: SRF | 6.86970 | transfac_public-M00215 logo | RCC of transfac_public-M00215 | link | link | PWMs |
| 32 | transfac_pro-M01164<br>Description: P$SQUA_01 | 6.81799 | transfac_pro-M01164 logo | RCC of transfac_pro-M01164 | link | link | PWMs |
| 33 | jaspar-PB0078.1<br>Description: Srf_1<br>Possible TFs: SRF | 6.61440 | jaspar-PB0078.1 logo | RCC of jaspar-PB0078.1 | link | link | PWMs |
| 34 | swissregulon-SRF.p3<br>Description: SRF<br>Possible TFs: SRF | 6.56596 | swissregulon-SRF.p3 logo | RCC of swissregulon-SRF.p3 | link | link | PWMs |
| 35 | jaspar-PF0033.1<br>Description: YTATTTTNR | 6.32414 | jaspar-PF0033.1 logo | RCC of jaspar-PF0033.1 | link | link | PWMs |
| 36 | homer-M00571<br>Description: Mef2/dmmpmm(Papatsenko)/fly<br>Possible TFs: MEF2C, MEF2B, MEF2A, MEF2BNB-MEF2B, MEF2D | 6.20650 | homer-M00571 logo | RCC of homer-M00571 | link | link | PWMs |
| 37 | yetfasco-1448<br>Description: YBR182C<br>Possible TFs: MEF2C, MEF2B, MEF2A, MEF2BNB-MEF2B, MEF2D | 6.18137 | yetfasco-1448 logo | RCC of yetfasco-1448 | link | link | PWMs |
| 38 | jaspar-MA0082.1<br>Description: squamosa | 6.16535 | jaspar-MA0082.1 logo | RCC of jaspar-MA0082.1 | link | link | PWMs |
| 39 | transfac_pro-M01257<br>Description: V$SRF_02<br>Possible TFs: SRF | 6.02622 | transfac_pro-M01257 logo | RCC of transfac_pro-M01257 | link | link | PWMs |

| # | Feature | NES | Logo | Recovery Curve | Candidate targets | All regions in top 20000 | Databa |
|---|---|---|---|---|---|---|---|
| 40 | factorbook-SRF<br>Description: SRF<br>Possible TFs: SRF | *5.97596* | factorbook-SRF logo | RCC of factorbook-SRF | link | link | PWMs |
| 41 | transfac_public-M00392<br>Description: P$AGL3_01 | *5.82555* | transfac_public-M00392 logo | RCC of transfac_public-M00392 | link | link | PWMs |
| 42 | transfac_public-M00393<br>Description: P$AGL3_02 | *5.79823* | transfac_public-M00393 logo | RCC of transfac_public-M00393 | link | link | PWMs |
| 43 | swissregulon-TBP.p2<br>Description: TBP<br>Possible TFs: TBP | *5.74797* | swissregulon-TBP.p2 logo | RCC of swissregulon-TBP.p2 | link | link | PWMs |
| 44 | taipale-NNMCCATATAWGGKNN-SRF-full<br>Description: NNMCCATATAWGGKNN-SRF-full | *5.74397* | taipale-NNMCCATATAWGGKNN-SRF-full logo | RCC of taipale-NNMCCATATAWGGKNN-SRF-full | link | link | PWMs |
| 45 | transfac_public-M00152<br>Description: V$SRF_01<br>Possible TFs: SRF | *5.68096* | transfac_public-M00152 logo | RCC of transfac_public-M00152 | link | link | PWMs |
| 46 | transfac_pro-M01304<br>Description: V$SRF_03<br>Possible TFs: SRF | *5.57316* | transfac_pro-M01304 logo | RCC of transfac_pro-M01304 | link | link | PWMs |
| 47 | stark-STATAWAWR<br>Description: STATAWAWR | *5.49595* | stark-STATAWAWR logo | RCC of stark-STATAWAWR | link | link | PWMs |
| 48 | homer-M00114<br>Description: MyoG(HLH)/C2C12-MyoG-ChIP-Seq(GSE36024)/Homer<br>Possible TFs: MYOG | *5.47956* | homer-M00114 logo | RCC of homer-M00114 | link | link | PWMs |
| 49 | taipale-MCCATATAWGGN-SRF-DBD<br>Description: MCCATATAWGGN-SRF-DBD | *5.45480* | taipale-MCCATATAWGGN-SRF-DBD logo | RCC of taipale-MCCATATAWGGN-SRF-DBD | link | link | PWMs |
| 50 | homer-M00830<br>Description: TBP(- | *5.38341* | homer-M00830 logo | RCC of homer-M00830 | link | link | PWMs |
| 51 | homer-M01793<br>Description: RLM1(MacIsaac)/Yeast | *5.38123* | homer-M01793 logo | RCC of homer-M01793 | link | link | PWMs |
| 52 | jaspar-PF0051.1<br>Description: TATAAA | *5.30147* | jaspar-PF0051.1 logo | RCC of jaspar-PF0051.1 | link | link | PWMs |
| 53 | transfac_pro-M00810<br>Description: V$SRF_Q4<br>Possible TFs: SRF | *5.29564* | transfac_pro-M00810 logo | RCC of transfac_pro-M00810 | link | link | PWMs |
| 54 | transfac_pro-M01062<br>Description: P$AGL2_02 | *5.25813* | transfac_pro-M01062 logo | RCC of transfac_pro-M01062 | link | link | PWMs |
| 55 | yetfasco-1310<br>Description: YPL089C<br>Possible TFs: MEF2C, MEF2B, MEF2A, MEF2BNB-MEF2B, MEF2D | *5.23300* | yetfasco-1310 logo | RCC of yetfasco-1310 | link | link | PWMs |
| 56 | transfac_pro-M00406<br>Description: V$HMEF2_Q6<br>Possible TFs: MEF2A | *5.19585* | transfac_pro-M00406 logo | RCC of transfac_pro-M00406 | link | link | PWMs |
| 57 | stark-STATAWAWRSVVV<br>Description: STATAWAWRSVVV | *5.11209* | stark-STATAWAWRSVVV logo | RCC of stark-STATAWAWRSVVV | link | link | PWMs |
| 58 | transfac_pro-M00922<br>Description: V$SRF_Q5_01<br>Possible TFs: SRF | *5.10517* | transfac_pro-M00922 logo | RCC of transfac_pro-M00922 | link | link | PWMs |
| 59 | factorbook-TBP<br>Description: TBP<br>Possible TFs: TBP, POLR2A, TAF1 | *4.95657* | factorbook-TBP logo | RCC of factorbook-TBP | link | link | PWMs |
| 60 | transfac_pro-M00403<br>Description: V$AMEF2_Q6<br>Possible TFs: MEF2A | *4.80907* | transfac_pro-M00403 logo | RCC of transfac_pro-M00403 | link | link | PWMs |
| 61 | yetfasco-625<br>Description: YPL089C<br>Possible TFs: MEF2C, MEF2B, MEF2A, MEF2BNB-MEF2B, MEF2D | *4.80033* | yetfasco-625 logo | RCC of yetfasco-625 | link | link | PWMs |
| 62 | jaspar-MA0083.1<br>Description: SRF<br>Possible TFs: SRF | *4.65320* | jaspar-MA0083.1 logo | RCC of jaspar-MA0083.1 | link | link | PWMs |
| 63 | transfac_public-M00252<br>Description: V$TATA_01<br>Possible TFs: TBP, TBPL2 | *4.63462* | transfac_public-M00252 logo | RCC of transfac_public-M00252 | link | link | PWMs |
| 64 | jaspar-MA0108.1<br>Description: TBP | *4.62005* | jaspar-MA0108.1 logo | RCC of jaspar-MA0108.1 | link | link | PWMs |
| 65 | tfdimers-MD00570<br>Description: NeuroD_GR | *4.36257* | tfdimers-MD00570 logo | RCC of tfdimers-MD00570 | link | link | PWMs |

| # | Feature | NES | Logo | Recovery Curve | Candidate targets | All regions in top 20000 | Databa |
|---|---|---|---|---|---|---|---|
| 66 | transfac_public-M00186<br>Description: V$SRF_Q6<br>Possible TFs: SRF | 4.33671 | transfac_public-M00186 logo | RCC of transfac_public-M00186 | link | link | PWMs |
| 67 | homer-M00353<br>Description: sna/dmmpmm(Bergman)/fly<br>Possible TFs: SNAI2, SNAI3, SNAI1 | 4.25877 | homer-M00353 logo | RCC of homer-M00353 | link | link | PWMs |
| 68 | yetfasco-2056<br>Description: YER148W<br>Possible TFs: TBP, TBPL1, TBPL2 | 4.22854 | yetfasco-2056 logo | RCC of yetfasco-2056 | link | link | PWMs |
| 69 | transfac_public-M00060<br>Description: I$SN_01<br>Possible TFs: SNAI2, SNAI3, SNAI1 | 4.18229 | transfac_public-M00060 logo | RCC of transfac_public-M00060 | link | link | PWMs |
| 70 | jaspar-PF0121.1<br>Description: CCAWWNAAGG | 4.00456 | jaspar-PF0121.1 logo | RCC of jaspar-PF0121.1 | link | link | PWMs |
| 71 | tiffin-TIFDMEM0000083<br>Description: TIFDMEM0000083 | 3.98963 | tiffin-TIFDMEM0000083 logo | RCC of tiffin-TIFDMEM0000083 | link | link | PWMs |
| 72 | jaspar-PF0015.1<br>Description: CAGCTG<br>Possible TFs: MYOG | 3.98817 | jaspar-PF0015.1 logo | RCC of jaspar-PF0015.1 | link | link | PWMs |
| 73 | homer-M00112<br>Description: Myf5(bHLH)/GM-Myf5-ChIP-Seq(GSE24852)/Homer<br>Possible TFs: MYF5 | 3.95321 | homer-M00112 logo | RCC of homer-M00112 | link | link | PWMs |
| 74 | homer-M00193<br>Description: TATA-Box(TBP)/Promoter/Homer<br>Possible TFs: TBP | 3.86580 | homer-M00193 logo | RCC of homer-M00193 | link | link | PWMs |
| 75 | transfac_pro-M03135<br>Description: N$HLH2HLH10_01 | 3.86471 | transfac_pro-M03135 logo | RCC of transfac_pro-M03135 | link | link | PWMs |
| 76 | jaspar-PF0173.1<br>Description: YWATTWNNRGCT | 3.82720 | jaspar-PF0173.1 logo | RCC of jaspar-PF0173.1 | link | link | PWMs |
| 77 | transfac_public-M00471<br>Description: V$TBP_01<br>Possible TFs: TBP | 3.81554 | transfac_public-M00471 logo | RCC of transfac_public-M00471 | link | link | PWMs |
| 78 | homer-M00234<br>Description: TATA-box/Drosophila-Promoters/Homer | 3.80243 | homer-M00234 logo | RCC of homer-M00234 | link | link | PWMs |
| 79 | transfac_pro-M01061<br>Description: P$AGL2_01 | 3.77584 | transfac_pro-M01061 logo | RCC of transfac_pro-M01061 | link | link | PWMs |
| 80 | transfac_pro-M02100<br>Description: V$MYOD_Q6_02 | 3.76565 | transfac_pro-M02100 logo | RCC of transfac_pro-M02100 | link | link | PWMs |
| 81 | transfac_pro-M02781<br>Description: V$MYF6_03<br>Possible TFs: MYF6 | 3.70628 | transfac_pro-M02781 logo | RCC of transfac_pro-M02781 | link | link | PWMs |
| 82 | jaspar-CN0169.1<br>Description: LM169 | 3.68043 | jaspar-CN0169.1 logo | RCC of jaspar-CN0169.1 | link | link | PWMs |
| 83 | selexconsensus-bin<br>Description: bin<br>Possible TFs: FOXQ1, FOXL2, FOXF1, FOXF2, FOXL1 | 3.57554 | selexconsensus-bin logo | RCC of selexconsensus-bin | link | link | PWMs |
| 84 | homer-M00172<br>Description: Six1(Homeobox)/Myoblast-Six1-ChIP-Chip(GSE20150)/Homer<br>Possible TFs: SIX1 | 3.50160 | homer-M00172 logo | RCC of homer-M00172 | link | link | PWMs |
| 85 | transfac_pro-M00319<br>Description: V$MEF3_B | 3.45207 | transfac_pro-M00319 logo | RCC of transfac_pro-M00319 | link | link | PWMs |
| 86 | transfac_pro-M01582<br>Description: P$AGL9_01 | 3.44078 | transfac_pro-M01582 logo | RCC of transfac_pro-M01582 | link | link | PWMs |
| 87 | jaspar-PF0035.1<br>Description: GCANCTGNY | 3.42439 | jaspar-PF0035.1 logo | RCC of jaspar-PF0035.1 | link | link | PWMs |
| 88 | flyfactorsurvey-sna_SOLEXA_5_FBgn0003448<br>Description: FBgn0003448(sna)<br>Possible TFs: SNAI2, SNAI3, SNAI1 | 3.40436 | flyfactorsurvey-sna_SOLEXA_5_FBgn0003448 logo | RCC of flyfactorsurvey-sna_SOLEXA_5_FBgn0003448 | link | link | PWMs |
| 89 | transfac_public-M00006<br>Description: V$MEF2_01<br>Possible TFs: MEF2A | 3.39016 | transfac_public-M00006 logo | RCC of transfac_public-M00006 | link | link | PWMs |
| 90 | elemento-AACAGCTG<br>Description: AACAGCTG | 3.34318 | elemento-AACAGCTG logo | RCC of elemento-AACAGCTG | link | link | PWMs |
| 91 | transfac_pro-M02024<br>Description: V$MEF2A_Q6<br>Possible TFs: MEF2A | 3.33043 | transfac_pro-M02024 logo | RCC of transfac_pro-M02024 | link | link | PWMs |

| # | Feature | NES | Logo | Recovery Curve | Candidate targets | All regions in top 20000 | Databa |
|---|---|---|---|---|---|---|---|
| 92 | transfac_pro-M02119<br>Description: V$T3RBETA_Q6_01<br>Possible TFs: THRB | *3.31732* | transfac_pro-M02119 logo | RCC of transfac_pro-M02119 | link | link | PWMs |
| 93 | encode-UW.Motif.0086<br>Description: UW.Motif.0086 | *3.29037* | encode-UW.Motif.0086 logo | RCC of encode-UW.Motif.0086 | link | link | PWMs |
| 94 | transfac_pro-M00949<br>Description: P$AGL15_01 | *3.25905* | transfac_pro-M00949 logo | RCC of transfac_pro-M00949 | link | link | PWMs |
| 95 | homer-M00256<br>Description: HLH-1(bHLH)/cElegans-Embryo-HLH1-ChIP-Seq(modEncode)/Homer | *3.25868* | homer-M00256 logo | RCC of homer-M00256 | link | link | PWMs |
| 96 | flyfactorsurvey-Fer3_da_SANGER_5_FBgn0037937<br>Description: FBgn0037937(Fer3)<br>Possible TFs: FERD3L | *3.24703* | flyfactorsurvey-Fer3_da_SANGER_5_FBgn0037937 logo | RCC of flyfactorsurvey-Fer3_da_SANGER_5_FBgn0037937 | link | link | PWMs |
| 97 | hdpi-MEIS3<br>Description: MEIS3<br>Possible TFs: MEIS3 | *3.24667* | hdpi-MEIS3 logo | RCC of hdpi-MEIS3 | link | link | PWMs |
| 98 | jaspar-MA0386.1<br>Description: TBP<br>Possible TFs: TBP, TBPL1, TBPL2 | *3.24557* | jaspar-MA0386.1 logo | RCC of jaspar-MA0386.1 | link | link | PWMs |
| 99 | transfac_public-M00233<br>Description: V$MEF2_04<br>Possible TFs: MEF2A | *3.21680* | transfac_public-M00233 logo | RCC of transfac_public-M00233 | link | link | PWMs |
| 100 | yetfasco-798<br>Description: YER148W<br>Possible TFs: TBP, TBPL1, TBPL2 | *3.21571* | yetfasco-798 logo | RCC of yetfasco-798 | link | link | PWMs |
| 101 | hdpi-JDP2<br>Description: JDP2<br>Possible TFs: JDP2 | *3.19167* | hdpi-JDP2 logo | RCC of hdpi-JDP2 | link | link | PWMs |
| 102 | transfac_pro-M00713<br>Description: F$TBP_Q6<br>Possible TFs: TBP, TBPL1, TBPL2 | *3.18657* | transfac_pro-M00713 logo | RCC of transfac_pro-M00713 | link | link | PWMs |
| 103 | transfac_public-M00184<br>Description: V$MYOD_Q6<br>Possible TFs: MYOD1 | *3.17638* | transfac_public-M00184 logo | RCC of transfac_public-M00184 | link | link | PWMs |
| 104 | flyfactorsurvey-ato_da_SANGER_10_FBgn0010433<br>Description: FBgn0010433(ato)<br>Possible TFs: NEUROD2, NEUROD1, NEUROD6, NEUROD4 | *3.11191* | flyfactorsurvey-ato_da_SANGER_10_FBgn0010433 logo | RCC of flyfactorsurvey-ato_da_SANGER_10_FBgn0010433 | link | link | PWMs |
| 105 | encode-UW.Motif.0005<br>Description: UW.Motif.0005 | *3.10135* | encode-UW.Motif.0005 logo | RCC of encode-UW.Motif.0005 | link | link | PWMs |
| 106 | stark-GTATNWATA<br>Description: GTATNWATA | *3.09297* | stark-GTATNWATA logo | RCC of stark-GTATNWATA | link | link | PWMs |
| 107 | homer-M00270<br>Description: TATA-box/SacCer-Promoters/Homer | *3.08387* | homer-M00270 logo | RCC of homer-M00270 | link | link | PWMs |

© 2015 KULeuven | Authors: Gert Hulselmans & Hana Imrichova | Web design (CSS) by Andreas Viklund.
