# Supplementary figures and images for: Systems Biology Reveals NR2F6 and TGFB1 as Key Regulators of Feed Efficiency in Beef Cattle
Source: Front Genet. 2019 Mar 22;10:230. doi: 10.3389/fgene.2019.00230 (PMC6439317; doi:10.3389/fgene.2019.00230)

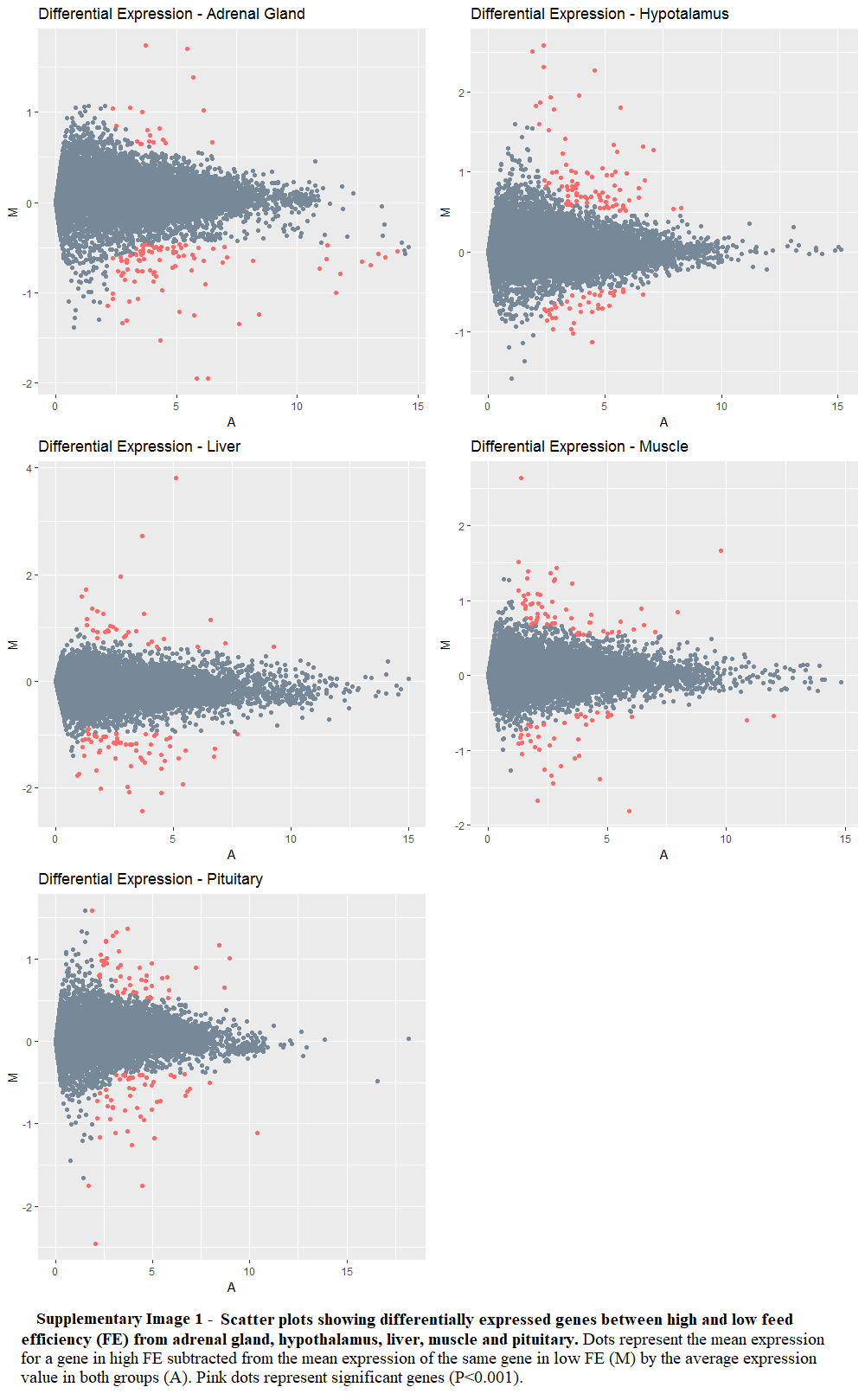

Supplement: Supplementary file 14 [file Image_1.PNG]

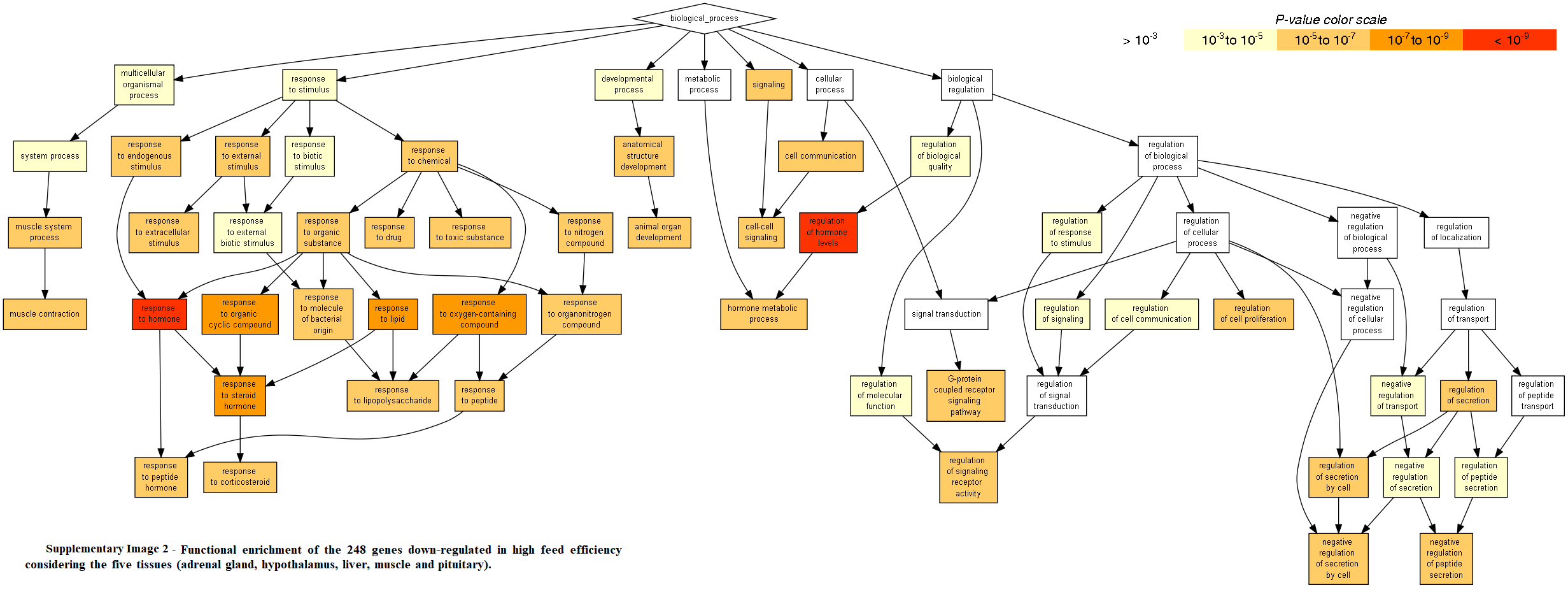

Supplement: Supplementary file 15 [file Image_2.PNG]

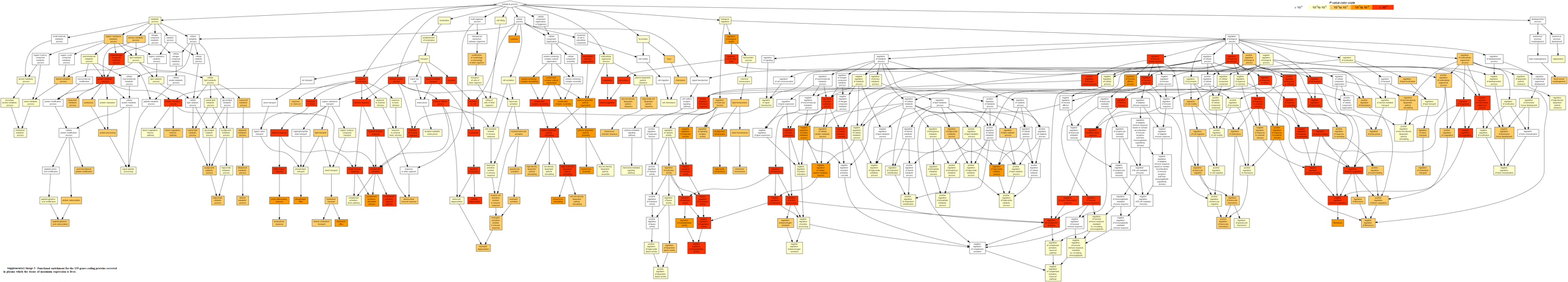

Supplement: Supplementary file 16 [file Image_3.PNG]

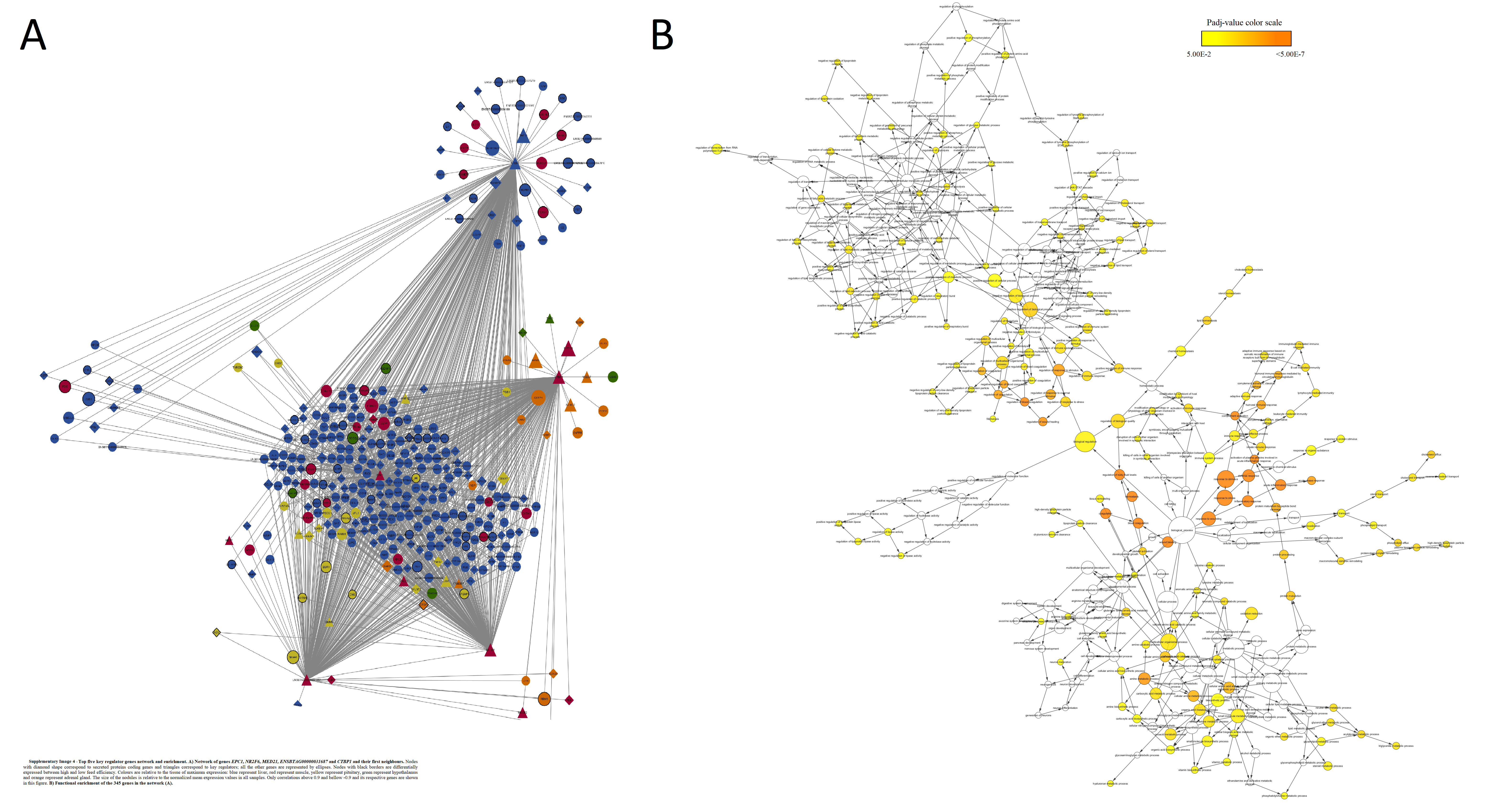

Supplement: Supplementary file 17 [file Image_4.PNG]

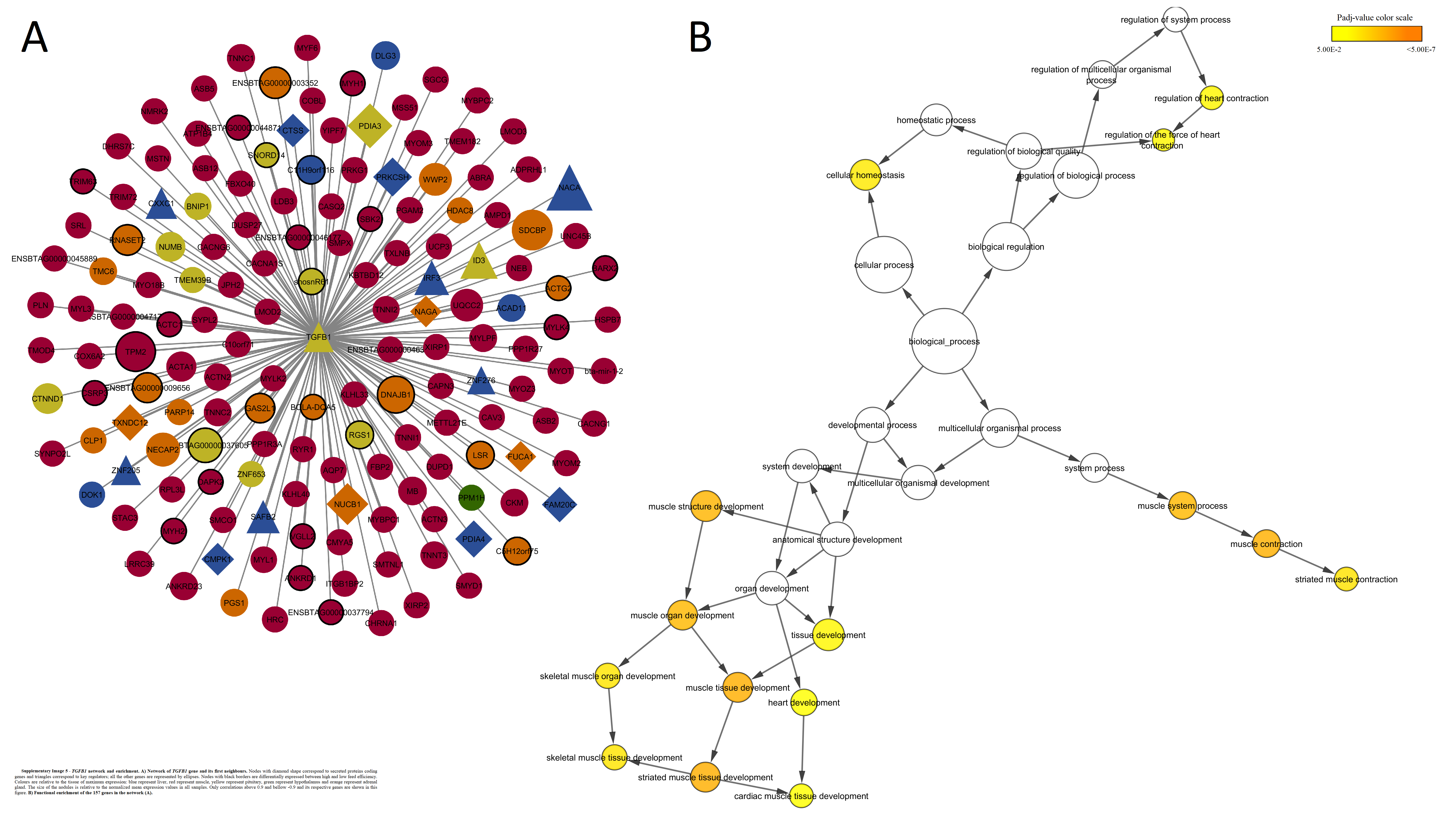

Supplement: Supplementary file 18 [file Image_5.PNG]

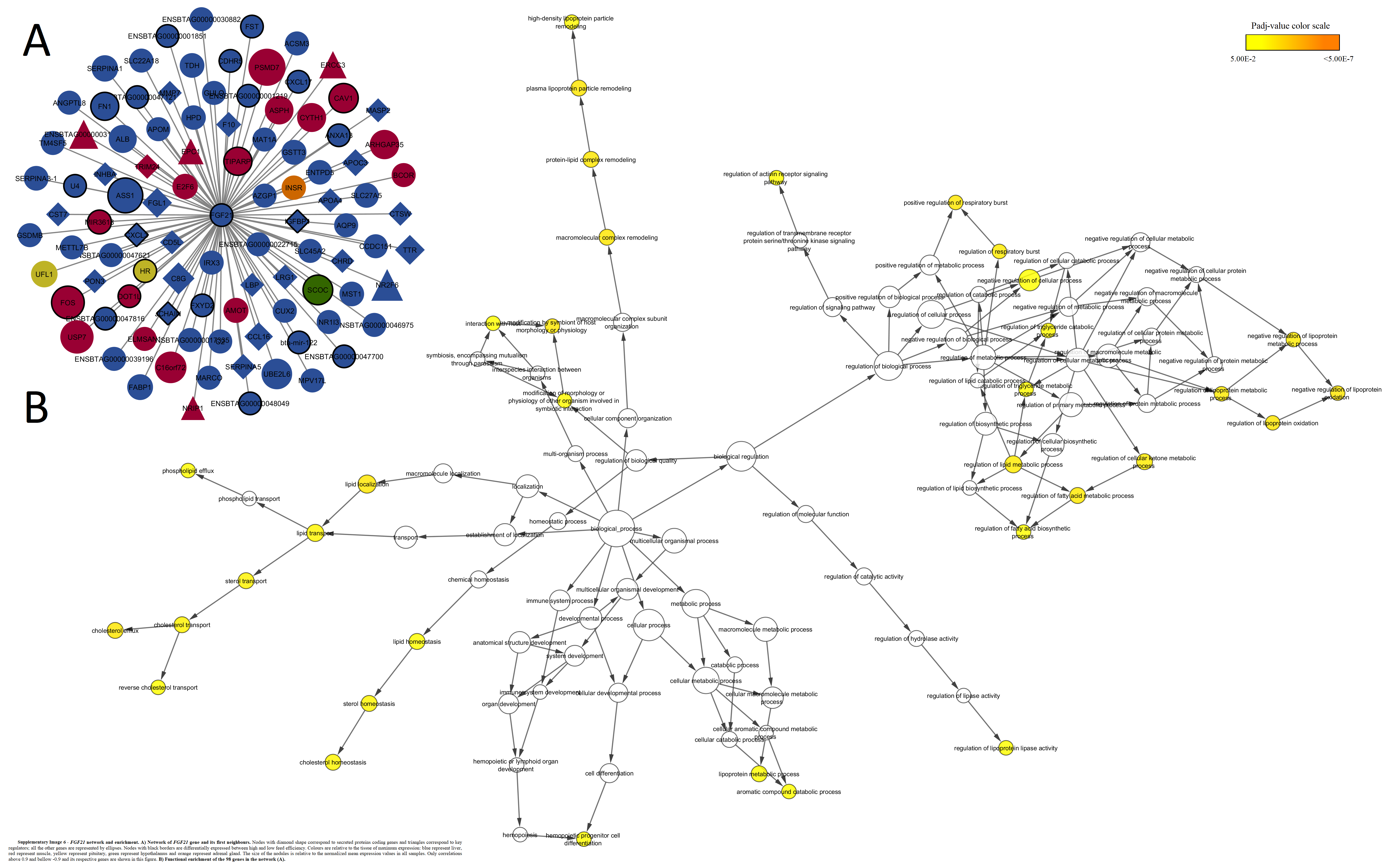

Supplement: Supplementary file 19 [file Image_6.PNG]

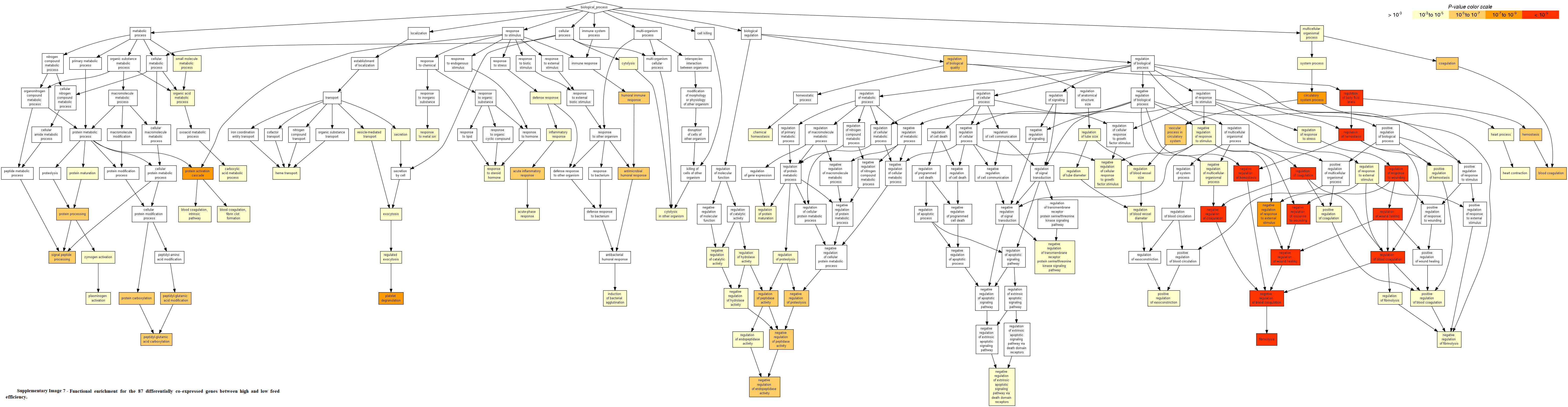

Supplement: Supplementary file 20 [file Image_7.PNG]
